# Weak power frequency magnetic fields induce microtubule cytoskeleton reorganization depending on the epidermal growth factor receptor and the calcium related signaling

**DOI:** 10.1371/journal.pone.0205569

**Published:** 2018-10-12

**Authors:** Xia Wu, Juan Du, Weitao Song, Meiping Cao, Shude Chen, Ruohong Xia

**Affiliations:** 1 Physics Department, East China Normal University, Shanghai, China; 2 State Key Laboratory of Precision Spectroscopy, East China Normal University, Shanghai, China; Hungarian Academy of Sciences, HUNGARY

## Abstract

We have shown previously that a weak 50 Hz magnetic field (MF) invoked the actin-cytoskeleton, and provoked cell migration at the cell level, probably through activating the epidermal growth factor receptor (EGFR) related motility pathways. However, whether the MF also affects the microtubule (MT)-cytoskeleton is still unknown. In this article, we continuously investigate the effects of 0.4 mT, 50 Hz MF on the MT, and try to understand if the MT effects are also associated with the EGFR pathway as the actin-cytoskeleton effects were. Our results strongly suggest that the MF effects are similar to that of EGF stimulation on the MT cytoskeleton, showing that 1) the MF suppressed MT in multiple cell types including PC12 and FL; 2) the MF promoted the clustering of the EGFR at the protein and the cell levels, in a similar way of that EGF did but with higher sensitivity to PD153035 inhibition, and triggered EGFR phosphorylation on sites of Y1173 and S1046/1047; 3) these effects were strongly depending on the Ca^2+^ signaling through the L-type calcium channel (LTCC) phosphorylation and elevation of the intracellular Ca^2+^ level. Strong associations were observed between EGFR and the Ca^2+^ signaling to regulate the MF-induced-reorganization of the cytoskeleton network, via phosphorylating the signaling proteins in the two pathways, including a significant MT protein, tau. These results strongly suggest that the MF activates the overall cytoskeleton in the absence of EGF, through a mechanism related to both the EGFR and the LTCC/Ca^2+^ signaling pathways.

## Introduction

The cell motility depends on the transformation and reorganization of the cytoskeleton network, which mainly consists of actin filaments (F-actin), microtubules (MT), and intermediate filaments. In stationary state, cells usually have obvious thick stress fiber bundles across cell centers, polarized MT distributed from cell center to periphery, and focal adhesions (FA) scattered all over the cell; while in migrating cells, the cytoskeleton is reorganized with F-actin much thinner in cell centers while denser in lamellipodia, MT scarcely reaching cell periphery, and FA more in leading edge and less in rear direction [1, 2].

The actin cytoskeleton transformation is the main force to drive cell motility, which is usually induced by activities of epithelial growth factor receptors (EGFRs) initiated actin turnover, and results in protrusional organelle spreading in cell front. The processes rely on the EGFR-Protein kinase C (PKC)- mitogen-activated protein kinase (also called extracellular signal-regulated kinases, MAPK/Erk) pathways [3–5]. It was well known that epithelial growth factor (EGF), the ligand of EGFR, induces cell migration in multiple normal and tumorous cell lines [5, 6] through overall activation of cytoskeleton network of actin, MT, and FA *etc*. [7–9]. We previously reported that a 30-min exposure of 0/1-0.5 mili Tesla (mT) power frequency magnetic field (MF) induced morphological and cytoskeletal changes in different cell lines [10–12]. In a way similar to EGF stimulation, a 30 min, 0.4 mT MF specifically upgraded cell migration, activated actin-cytoskeleton, induced a weakened F-actin network with denser filopodia and lamellipodia in leading edge, and re-distributed FA at cell level [13]. MF also induced responses at the protein level on signaling molecules such as F-actin nucleation protein Arp2/3, F-actin stabilizer protein fascin and MLC, as well as FA component vinculin *etc*. [13]. Nevertheless, it is still unclear if and how another cytoskeleton major component, MT, is affected by MF.

The MT forms spindle, centriole, and flagella, implicating in cellular transportation, motility, and maintenance of the cell shape *etc*.. MT tips extend to the cell edge all around the periphery in stationary cells, but rarely penetrate the protrusion structure (like lamellipodium) in the leading edge in migrating cells [2]. In eukaryotic cells, MT emanates from microtubule-organizing center (MTOC), which is the MT nucleation center located close to the nucleus and undergoes reorientation towards the migration leading edge [2]. The MTOC is usually centrosome, but non-centrosome MTOC sites also widely exist in differentiated cells [14]. When MT depolymerization occurs, the fibers are sheared into short segments [15–17]. The MT dynamic instability participates in regulation of motility by coordinating F-actin transformation and FA renewal [2, 18]. MT possibly interacts with cortical actin network to determine stationary or migratory state of the cell [2]. Especially, MT is important in neuron cells, where it forms neural tubes, determines the position of axon, and participates in early neurite differentiation [19].

As another significant component of the cytoskeleton, it was well studied that MT is configured by numerous proteins bound to the assembled MT tubes, such as tau and other microtubule associated proteins (MAPs) [20]. Protein tau is under the regulation of EGFR through the subsequent activation of MAPK/Erk depending on PKC phosphorylation [21–23]. Upon phosphorylated, the tau protein is less favored to bind to MT tubes, resulting in MT instability [24]. Recent researches found that the Alzheimer’s disease (AD) is associated with abnormally phosphorylated tau and EGFR signaling in the neuron cells [25, 26]. Additionally, the PKC-dependent MAPK/Erk activation is also essential for neural differentiation [27, 28]. Thus, we are very curious if the MF would also influence the MT network via phosphorylating PKC and tau.

EGFR, playing a key role overall in cell motility and activation of cytoskeleton, is a 170 KD protype tyrosine kinase (RTK), mainly activated by EGF binding and other EGF agonists including transforming growth factor α (TGFα) [29]. EGFR protein consists of a large extracellular ligand-binding region (domain I-IV) [30]. Upon ligand stimulation, the domain I and III rearrange to place near enough to bind simultaneously to the same ligand [30]. This rearrangement indirectly facilitates domain II to program a deviation at the disulfide-bonded, resulting in an extended formation to contact with opposite domain I of the other monomer, termed exposure of dimerization arm [30]. The first step of EGFR activation is the ligand-induced dimerization, resulting in autophosphorylation at 5 tyrosine (Y) sites (Y992, Y1045, Y1068, Y1148 and Y1173), enabling a signal transduction cascade by phosphorylation of its substrates [31]. Phosphorylated Y1173 residue serves as a major docking site of EGFR [32, 33] for the Src homology and collagen (Shc) proteins, which involve in MAPK/Erk signaling as scaffold proteins [34]. EGFR Y1173 phosphorylation also activates MAPK/Erk pathway through mediating PKC phosphorylation via phospholipase Cγ (PLCγ) and diacyl glycerol (DAG) [35, 36]. By this manner, EGFR regulates the cell motility through the PKC-MAPK/Erk pathway. Meanwhile, phosphorylation on S1046/1047 desensitizes EGFR and decreases its affinity for ligand binding to recover the rest state [37]. We have previously revealed that the 0.4 mT 50 Hz MF alone induced EGFR clustering in solution of purified EGFR and in the membrane of Chinese hamster lung (CHL) cells in the absence of ligand binding, and this morphological change was blocked by the EGFR tyrosine kinase (TK) inhibitor PD153035 (PD) [38]. However, we do not know if these MF-effects are based on the physiological interaction among monomers and result in EGFR activation.

Calcium signaling also deeply participates in the EGFR induced cytoskeleton dynamics. EGFR signaling is reported to crosstalk with the calcium-signaling pathway. On one hand, the calcium receptors are able to transactivate EGFR [39, 40]. On the other hand, EGFR activation upon EGF binding triggers Ca^2+^ influx through the plasma ion channels such as the L-type Ca^2+^ channel (LTCC) [40–42]. Calmodulin (CaM) is the primary binding object of Ca^2+^, existing in cells usually in MARCKS sink [43]. However, CaM could not be released from this sink unless MARCKS is phosphorylated by PKC [43], which is activated upon EGFR activation [35, 36], as well as by Ca^2+^ binding to CaM [44]. CaM/Ca^2+^ complex binds to the Ca^2+^/calmodulin-dependent protein kinase II (CAMK II) and regulates its phosphorylation [45]. Importantly, both PKC and CaMKII could activate MAPK/Erk signaling [3, 46, 47], which is implicated in modulating the MT and F-actin networks through regulating proteins including tau [24], fascin [48], Arp2/3 [49], MLC and vinculin [50] etc.. Hence, an optimal intracellular Ca^2+^ level is required to regulate the actin [51, 52] and the MT [53] associated regulating proteins, thus to modulate the F-actin and MT networks, as well as to shift cell motility. Since MF induced cell migration and cytoskeleton reorganization [13], an elevated intracellular calcium level and enhanced activities of the calcium channel, CAMK II, CaM, and MARCKS by MF are expected.

Power frequency MF, ubiquitous in daily life, belongs to the extremely low frequency MF (ELF-MF). Whether exposure to weak ELF-MFs induces healthy problems in robust people has turned into an acute environmental health issue, and results in vast studies focusing on effect of the ELF-MFs, providing massive evidence and opinions conflicting with or supporting to each other. It was reported that the power frequency MF disturbed the ligand/receptor activities [54], promoted the cellular calcium intaking [55–58], and modulated the calcium channels [59]. It was also reported that EGFR activated cytoskeleton transition highly depended on a physiological Ca^2+^ level in cells, and the MF effects on the Ca^2+^ activities were similar to those of the EGF [40–42], suggesting the MF induced EGFR-cytoskeleton transitions involves the calcium mobilizing and signaling.

At the present stage, we do not have clear pictures to show, under the power frequency MF exposure, 1) the microcosmic and dynamic MF-induced-EGFR protein interactions to form clusters at molecule level; 2) based on the previous results on the actin-cytoskeleton, if and how the fields activate the MT cytoskeleton via the EGFR clustering; 3) if the Ca^2+^ signal pathway is involved in the MF-induced-cytoskeleton effect. These are the main objects we focused on in this paper.

## Results and discussions

### MF evoked the MT network into a migration state depending on EGFR and LTCC

#### MF depressed the microtubule network

**Fig 1:**
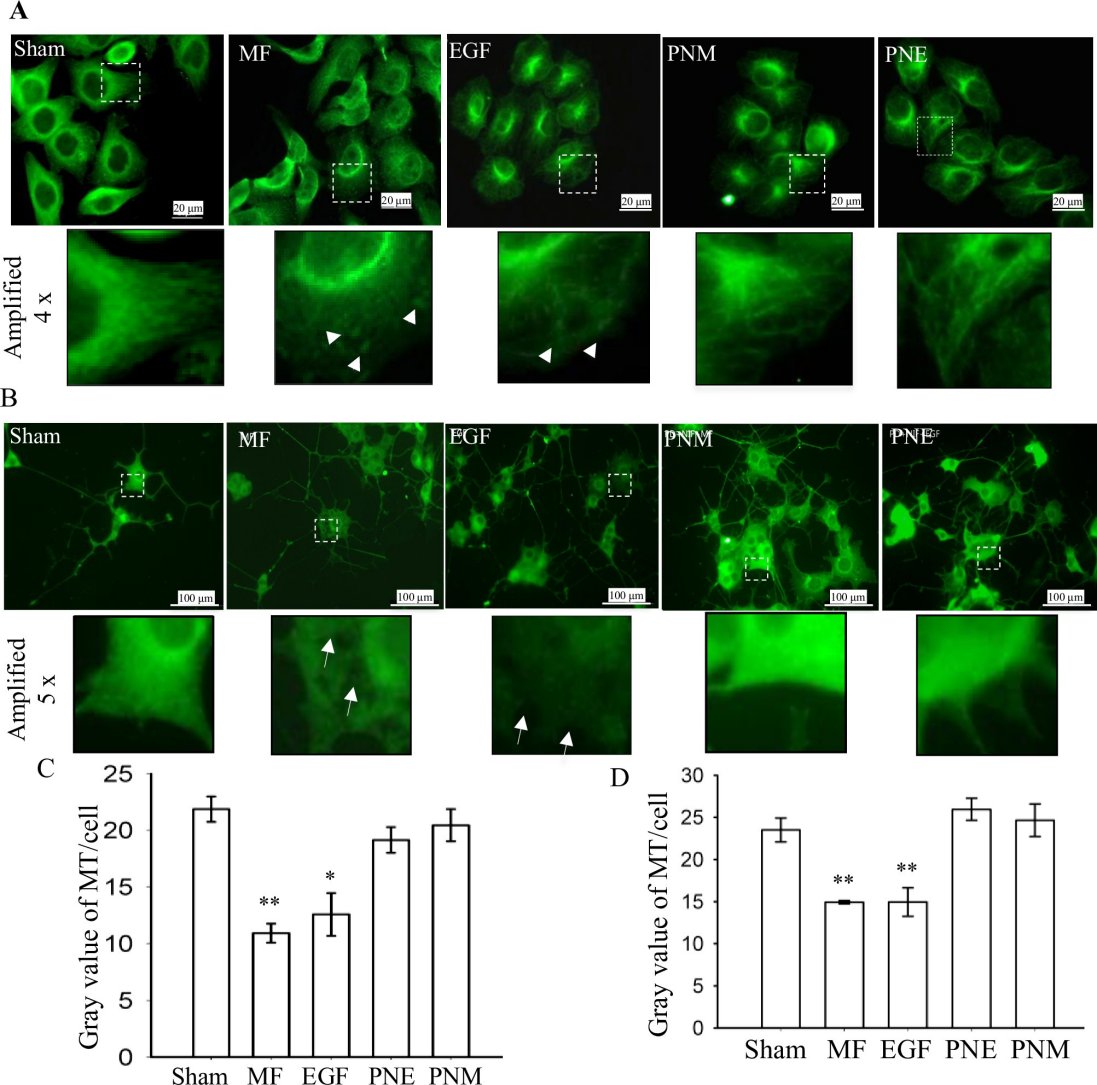
MF induced changes in microtubule cytoskeleton. MF induced changes in MT in FL cells (A) and in PC12 cells (B); and the average gray value / content of MT per cell was seen in C (FL) and D (PC12). Sham: sham exposure for 30 min; MF: 50 Hz 0.4 mT MF exposure for 30 min; EGF: 100 nM EGF treated for 30 min; PNM and PNE: pretreated with 1 μM PD for 2 h and 20 μM NIF for 40 min before 50 Hz 0.4 mT MF exposure (PNM) or EGF treatment (PNE) for 30 min. The horizontal bar represents 20 μm in A and 100 μm in B. The part in dashed square was further amplified, as shown in the panels below, by 4 x for FL and 5x for PC12 cells. In C and D: more than 50 cells were measured for each group (see details in S1 Table); * and **: p-value < 0.05 and < 0.01 when compared to Sham by Student’s t-test, respectively.

We previously reported that the 0.4 mili Tesla (mT), 50 Hz magnetic field (MF) exposure of 30 min altered the F-actin cytoskeleton, cell shape, and induced cell migration [13]. Hence, we suspected that the microtubule (MT) network, another main component of cytoskeleton, would also be shifted into a migration state by MF. To address this issue, β-tubulin, the MT monomer, was labeled to analyze the protein distribution and content level. The results confirmed that MT in stationary state [2] appeared as a clear network pattern, with pretty MT fibers emanating from the microtubule-organizing center (MTOC) ring around the nucleus in cell center and reaching the cell periphery in sham exposed FL cells (Fig 1A, Sham). However, after the MF exposure, the cell shape shifted, with nucleus away from cell center. The MT turned into an unstable and typically migrating status [60], with MTOC turning into half-ring shape and reoriented to face the migration leading edge, the MT intensity decayed in cell leading edge (lamellipodia), and fewer MT fibers reaching cell periphery (Fig 1A, MF). Instead, several bright spots appeared (arrow heads in Fig 1A, MF), possibly resembling depolymerized MT segments [15–17]. Meanwhile, it was found that, compared to the sham, the average MT content per cell decreased 50.01% upon MF exposure (Fig 1C). These MF effects were similar to those with EGF treatment (Fig 1A, EGF, and Fig 1C). Taken together, we showed here that MF could trigger MT into an instability state, possibly with higher speed of depolymerization and reassembly. As well known, it is the MT dynamics or its dynamic instability, but not the presence of MT per se, that affects the cell migration [60]. Moreover, we have demonstrated that both MF and EGF triggered cell motility in FL cells [13]. Therefore, here we have shown that the MF invoked FL cells into a ready-to-migrate or migrating state in a way similar to EGF stimulation.

MT plays an extremely key role in the function of neuron cells during formation of neural tubes, determination of the axon positions, differentiation of early neurites, and so on [61]. Therefore, we especially inspected the MF influence on the MT network in PC12 (pheochromocytoma 12) cells. The results showed that, similarly, the MTOC ring was intact and homogeneous in the sham-exposed cells, but broken and/or inhomogeneous in the MF or EGF treated cells (Fig 1B). Surprisingly, several “black hollows” of microtubules were observed in the cell body of MF and EGF treated cells (arrows in Fig 1B), with a loss of MT contents by 36.32% and 36.53%, respectively, when compared to sham (Fig 1D). Thus, similar to EGF, the MF is likely able to activate the turnover of MT-cytoskeleton system in both FL and PC12 cells, resulting in a weakened MT network. Moreover, the chronic exposure of MF was found to enhance the differentiation rate in the middle stage (S1 Fig), though the causality relationship between the effects of MF on MT and differentiation is unclear.

**Fig 2:**
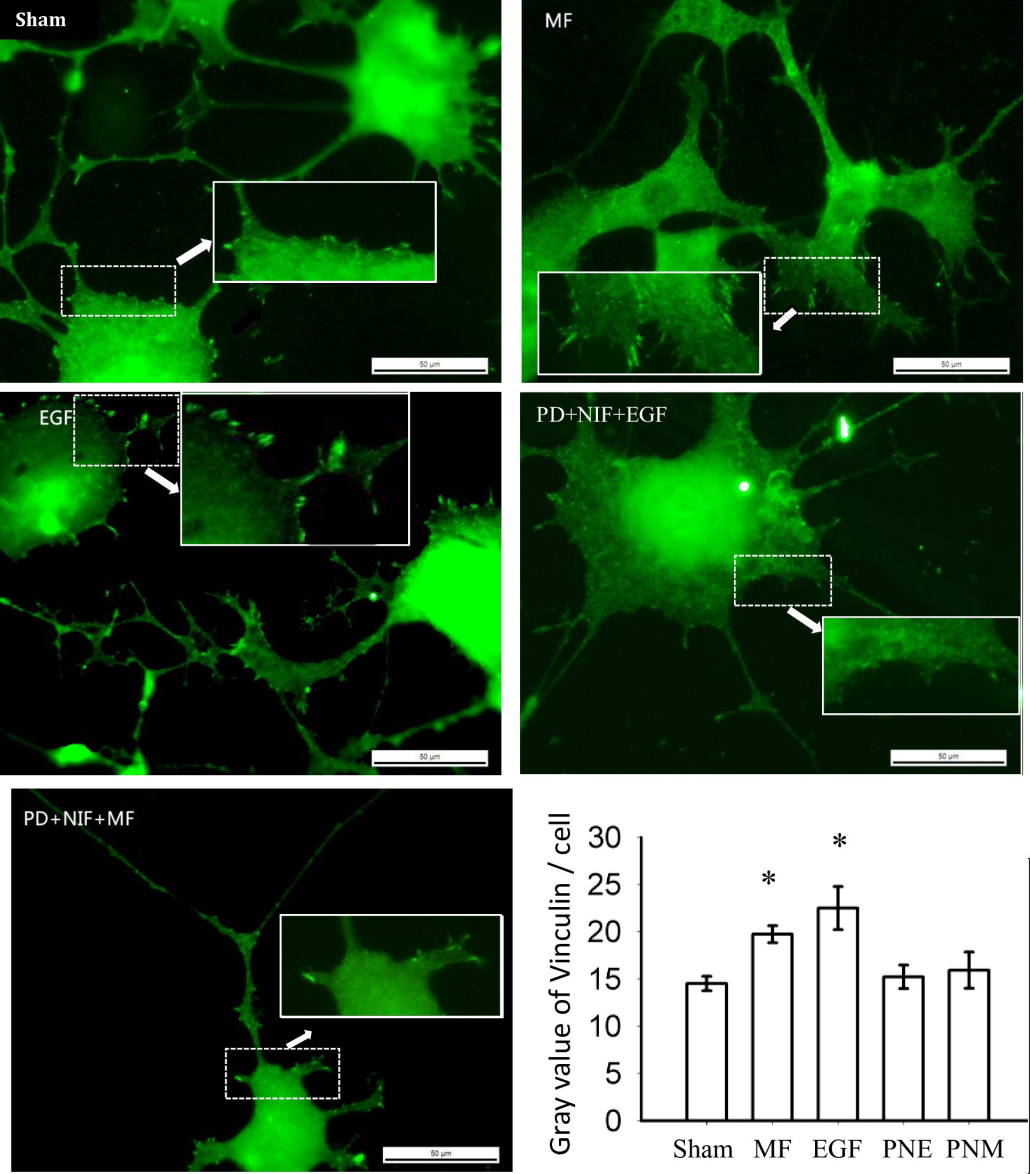
MF induced more focal adhesion in PC12 cells periphery. The groups were treated as described in Fig 1 and in Materials and Methods. The parts in dashed square were further amplified 2x, as indicated by the arrows. The horizontal bar represents 50 μm. The average gray value / content of vinculin per cell was quantified for each group within at least 50 cells (see details in S1 Table) and was shown in the histogram; *: p-value < 0.05 when compared with Sham by student’s t-test.

Motile cells are featured with focal adhesions (FA) denser in the leading edge and degraded in the rear, rather than homogenously distributed all over the cell body in the stationary state [2]. We previously observed that the FAs were formed and appeared in the lamellipodia in the leading edge of FL cells while MF triggering cell migration [13], possibly through proteins paxillin and FAK ([62]. In PC12 cells, again, much higher FA density unequally appeared in cell body periphery in the MF exposed cells, similar to the effects in the EGF treated cells (Fig 2). These results confirmed that the MF functions similarly to EGF stimulation to trigger PC12 cells prone into migration state, as it did with FLs [13].

#### The MF effects on the MT network may associate with activation of EGFR and LTCC

We have shown that PD inhibition of EGFR activation partially eliminated the depolymerization of stress fibers (F-actin) and the formation of protrusion structures induced by MF or EGF [13]. Therefore, besides the EGFR pathway, there must be some other signaling molecules involved in the mechanisms whereby MF exerted effects on cytoskeleton system. It was reported that calcium signaling also plays important roles in regulating cytoskeleton reorganization and cell migration [51, 58]. Hence, we investigated the MF effects on L-type calcium channel (LTCC), a typical calcium channel on cell membrane. When pretreating the FL cells with nifedipine (NIF) to inhibit LTCC alone, the MT and EGF induced F-actin depolymerization and morphology changes were also partially rescued (unpublished data in the lab and S2 Fig). When simultaneously blocking the activations of both EGFR by PD and LTCC by NIF, we found the MTOC ring was retained in FL after MF exposure (PNM, Fig 1A), and the “black hollows” (PNM, Fig 1B) and redistribution of FA (PNM, Fig 2) induced by MF disappeared in PC12, while the average content of MT per cell was recovered to the sham control level in both cell lines (Fig 1C and 1D). Similarly, the EGF induced MT changes and FA alterations were also diminished when EGFR and LTCC were simultaneously inhibited (PNE, Figs 1 and 2). Thus, it suggests that the MF or EGF induced effects on the MT system are related to both the EGFR and the LTCC signals. However, the function of the calcium signaling in the MF-induced MT instability was still unclear, and will be addressed in this paper later.

We previously showed that MF could induce FA redistribution, F-actin reorganization, and cell motility in FL cells in a way similar to EGF stimulation [13]. Here we showed that the MF exposure could induce MT instability in both FL and PC12 cells (Fig 1), and FA redistribution in PC12 cells (Fig 2), and all these effects could be almost fully eliminated by inhibition of both EGFR and LTCC. This reminisced the MF-induced EGFR clustering [38] and calcium intaking [55–58].Taken together, we demonstrated that MF could overall activate both F-actin and MT cytoskeleton system and FA in a mechanism depending on both EGFR and calcium signals, triggering cells into a migrating or ready-to-migrate state.

### MF induced redistribution, clustering, and activation of EGFRs

Since the MF-induced morphology transformation of the F-actin cytoskeleton system is related to the EGFR pathway [13], and the MT is also under the regulation of EGFR [8], does it mean the MF effects on the MT instability were initiated at the activation of the EGFR? To address this question, we examined the EGFR responses to MF exposure at both protein and cell levels.

#### MF enforced the EGFR clustering effect in the FL and PC12 cell lines

**Fig 3:**
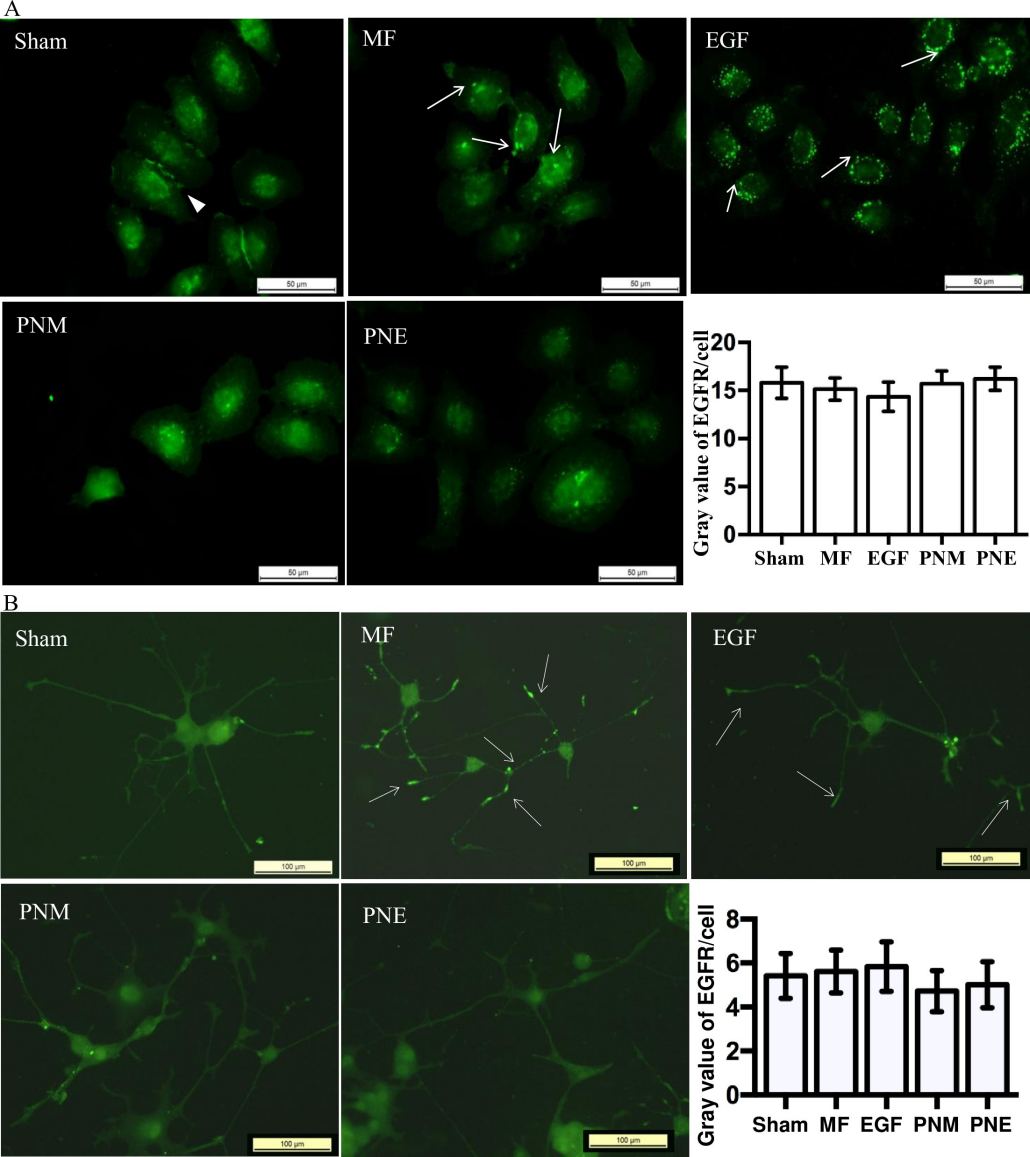
MF induced clustering and redistribution of EGFR. EGFR was labeled in green fluorescence in FL cells (A), and PC12 cells (B). The groups were treated as described in Fig 1 and in Materials and Methods. The horizontal bar represents 50 μm in A, and 100 μm in B, respectively. The average gray value /content of EGFR per cell was quantified in the bar chart, and more than 50 cells were measured for each group (see details in S1 Table), p-value > 0.05 when compared to the Sham by Student’s t-test. For all experiments, the repeat times were seen in the S1 Table.

The first step of EGFR activation is its dimerization/clustering [31], which is induced usually by extracellular ligands such as EGF, TGF, and so on [39], but could be also affected by many physical factors like MF [38]. We have found previously that MF induced the EGFR clusterization at both cell and protein levels [38]. In this work, as shown in Fig 3A and 3B, immunofluorescence detection revealed MF induced EGFR clustering in both FL and PC12 cells, as indicated by the bright green dots (arrows in Fig 3A and 3B, MF), which confirmed our previous observation that the MF induced EGFR clustering in CHL cells [38]. For PC12 cells, especially, the EGFR clusters emerged not only in the cell body, but also on the axons (Fig 3B, MF). Moreover, in the sham cells, the EGFR could be visualized all over the cell body (Fig 3, Sham), with sheet of proteins sometimes in FL cell periphery (arrowhead in Fig 3A, Sham), whilst upon MF exposure, the proteins were more concentrated around cell nucleus (Fig 3, MF), much fewer in cell periphery, interestingly except for axons in PC12 cells (Fig 3B, MF). Similar and stronger clustering effects were observed in EGF treated cells (Fig 3, EGF). Both MF and EGF did not exert significant influences on the total content of EGFR protein in both cell lines (Fig 3A and 3B, histograms). These results indicated that the MF induced a morphological EGFR redistribution and clustering in a manner similar but interior to EGF did.

Next, we tried to inspect the protein interaction process in a deeper look to give a microcosmic picture of EGFR clustering, by comparing the fluorescence resonance energy transfer (FRET) [63] signals which reflects EGFR clustering in cells and in solution of purified EGFR monomers treated with sham, MF and EGF (see details in the Materials and Methods).

**Fig 4:**
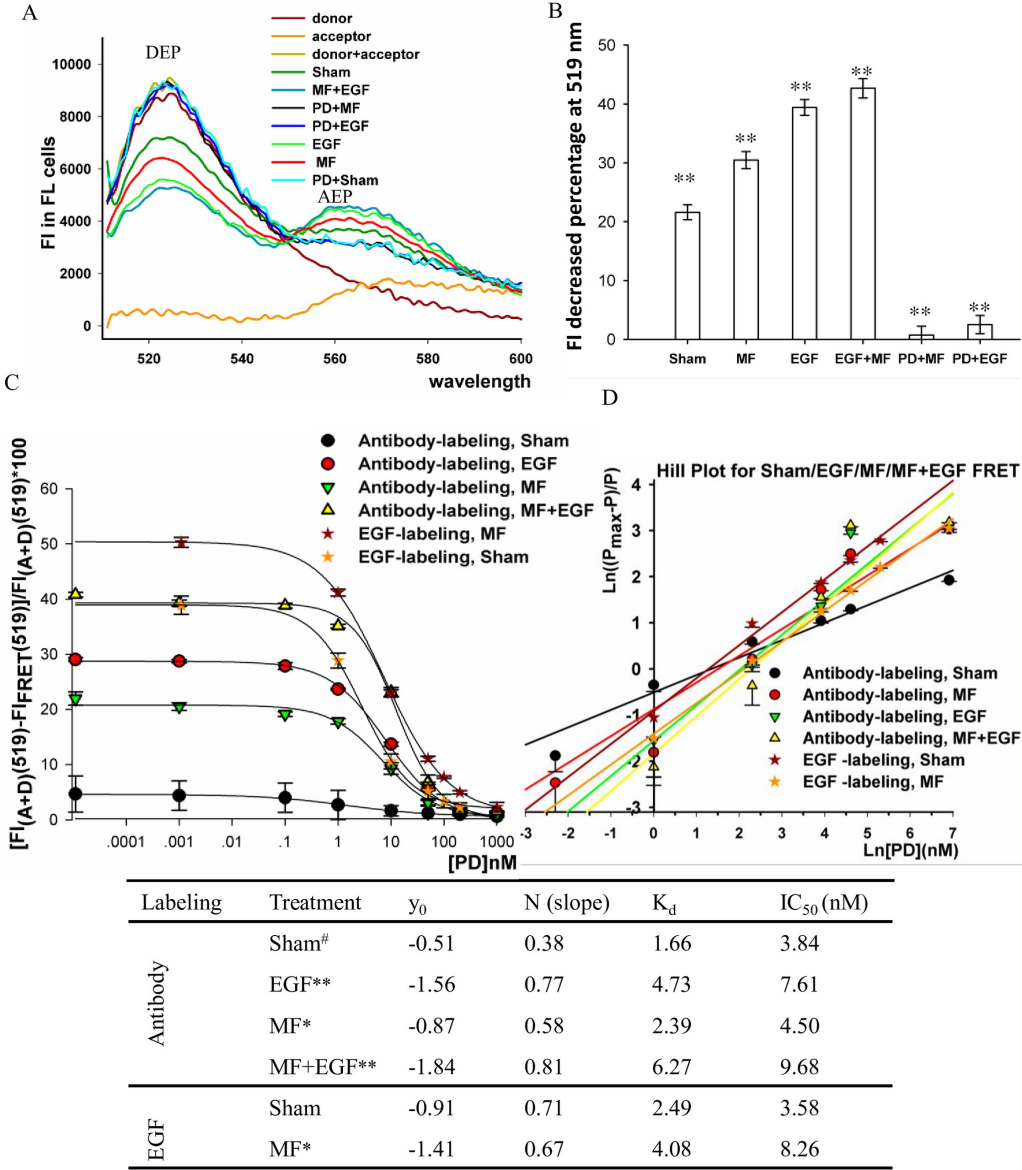
MF induced EGFR interaction. A: Fluorescence intensity of EGFR in differently treated FL cells. The plots represent the FI along wavelength in FRET groups of FL cells treated with sham exposure for 30 min (Sham), 0.4 mT 50 Hz MF for 30 min (MF), 100 nM EGF for 30 min while sham exposed (EGF) or 0.4 mT 50Hz MF exposed (MF+EGF), or pretreated with 1 μM PD for 2 h before 30 min treatment of 100 nM EGF (PD+EGF) or 0.4 mT 50 Hz MF (PD+MF). When cells were labeled with only donor dye (donor) or only acceptor dye (acceptor), the indicated treatment did not change the FI, thus only one single plot was shown as example; donor + acceptor: the mathematically sum of the FI in the donor and in the acceptor. DEP: Donor’s emission peak; AEP: Acceptor’s emission peak. B: Quantification of the FI decreases at donor emission peak from A. **: p-value < 0.01 in MF, EGF, and MF+EGF group when compared with Sham by Student’s t-test. C: The DEP FI decrease percentages in EGFR monomer solution with pretreatment of PD of a serial of concentration. D: Hill Plot analyses of the data in C; error bar was representative of Ln(mean-sd); more details seen in Materials and Methods. E: K_d_ and IC_50_ of PD inhibition to purified EGFR proteins upon different treatments by Hill plot; * and **: p-value < 0.05 and < 0.01, respectively, when compared to Sham with the same labeling; ^#^: the error bars in this group (shown in C) were so high that the extracted values might not reflect the real case. The repeat time of the experiments was listed in S1 Table.

The typical fluorescence intensity (FI) in cells along the wavelength was shown for each condition in Fig 4A. In this paper, we defined the FRET signal as the FI decrease at the donor emission peak (519 nm) in FRET group respective to donor+acceptor (sum of groups with donor alone or acceptor alone), which represents the intensity of energy exchange between donor dye and acceptor dye when the distance between the two molecules is close enough to a quantum scale. Therefore, the FRET signal strength indicates the quantitated amount of EGFR molecule interactions occurred. Obvious FRET signals were seen under the conditions of sham, MF, EGF, and MF+EGF at a degree of (21.58 ± 1.26)%, (30.47 ± 1.45)%, (39.42 ± 1.34)%, and (42.67 ± 1.64)%, respectively (Fig 4B), reflecting significant energy exchange enforcements under these conditions. The FRET signal was readily seen in the sham group, reflecting a control level of EGFR clustering occurred even in the absence of EGF in the serum starved FL cells, possibly due to EGFR clustering in a ligand-independent manner [64] or EGFR transactivation by other pathways [39, 40]. The increase of FRET signal in EGF treated cells was significantly stronger than that in the MF treated cells, indicating EGF had a higher efficiency on inducing the EGFR interaction than MF did (Fig 4B). The largest increase was observed in the EGF+MF group, though the difference compared to EGF alone was not large (Fig 4A and 4B), likely due to the possibility that 100 nM EGF stimulation triggered EGFR interaction to an almost saturated extent, so that additional MF exposure to the EGF treatment did not impact much further more. Nevertheless, these results implied that MF, even in present of EGF, is able to addictively enforce the EGFR monomers in cells to approach to a quantum-small distance between each other to form EGFR clusters.

#### PD inhibition of EGFR clustering is more efficient to MF than to EGF

It was known that EGF induced EGFR oligomerization results in EGFR activation [30, 31], and both EGFR oligomerization and activation could be blocked by PD153035 (PD) pretreatment on TK domain [38, 65]. Hence, it is interesting to investigate the effects of MF on the TK domain. In the previous work, we have seen that PD much inhibited the EGFR clustering in CHL cells and the cytoskeleton activation in FL cells in both MF and EGF treated samples [13, 38]. In this article, we found that in FL cell samples with 1000 nM PD pretreatment, the FRET signals were almost totally eliminated in the sham condition (Fig 4A). Similarly, the FRET signals were much depressed in both PD+MF and PD+EGF treated groups to a degree as subtle as (0.74 ± 1.50)% and (2.53 ± 1.55)%, respectively (Fig 4A and 4B). These results indicated that both the MF- and the EGF-induced EGFR clustering in cells was almost totally inhibited by the PD, suggesting that the MF induces EGFR clustering probably through a mechanism similar to EGF does, which is related to the TK domain [65]. The FRET signal was even lower in PD+MF than in PD+EGF (Fig 4B), but not significantly, indicating that PD inhibition to the MF-induced EGFR clustering is somehow likely more effective than to that of EGF (Fig 4B).

To further address this issue, FRET experiments were carried out with purified EGFR protein monomers in a protein suspension (see Materials and methods for details) pretreated with PD in a concentration dependent manner before exposure to MF or EGF. The results showed that the FRET signals were gradually depressed in a [PD] dependent manner in all conditions (Fig 4C), indicating that the EGFR clustering were overall constrained in the presence of PD. Further Hill Plot analyses (Fig 4D) allowed to extract the apparent dissociation constant K_d_ values of PD-EGFR interaction from the FRET vs. [PD] plot. It was found that the apparent K_d_ values were bigger in both MF group (2.39) and EGF group (4.43) than that in the sham (1.66) (Fig 4E). This result indicates that both MF and EGF play a role by counteracting to PD inhibition, and that the MF effects may target to the TK domain of EGFR. This is a first-hand evidence that the MF shifts the apparent affinity of the receptor to its inhibitor. However, the MF induced EGFR clustering was still more sensitive to PD inhibition than EGF induced, since the K_d_ or IC_50_ was smaller in MF group than in EGF group (Fig 4E). Taking all these results together, our evidence further confirmed the evidence that PD is able to block the MF triggered EGFR clustering, in a way similar but more efficient than it did to EGF induced clustering.

#### MF elevated the EGFR phosphorylation levels

**Fig 5:**
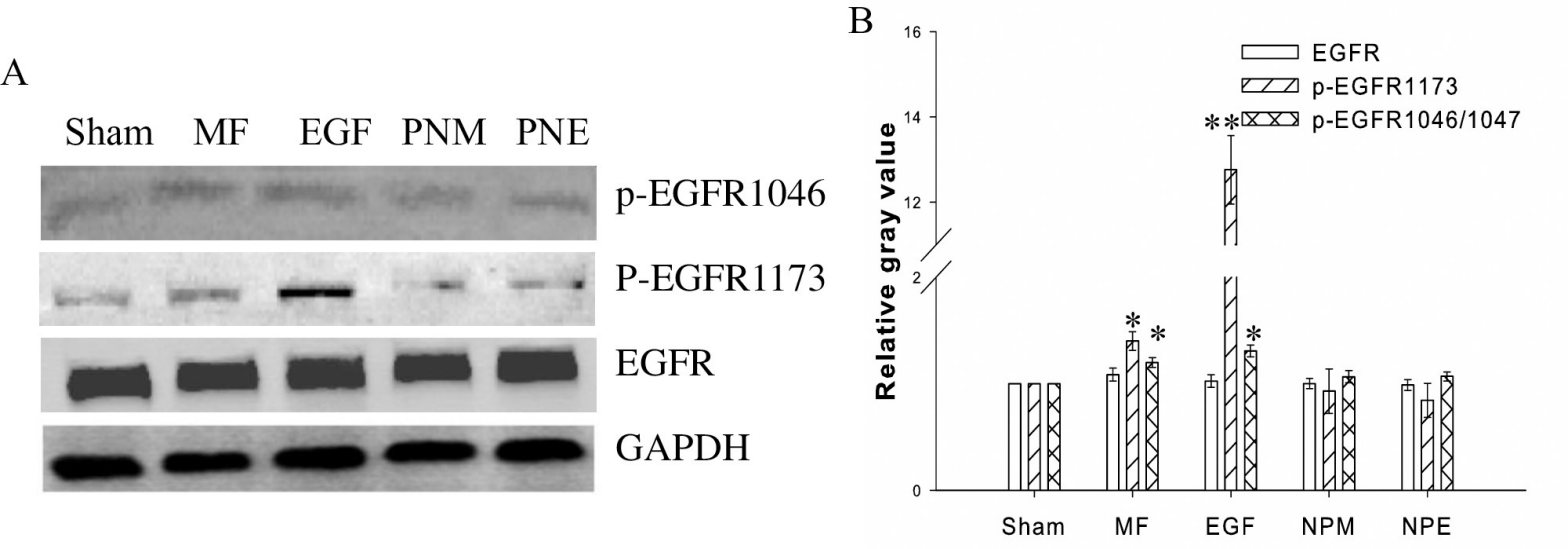
MF activated EGFR. A: Western blot of EGFR and EGFR phosphorylation at residue Tyr1173 and Ser1046; B: relative gray value/content of the proteins to the Sham groups after normalized with the GAPDH content, * and **: p-value < 0.05 and < 0.01 when compared with Sham by Student’s t-test, respectively. The groups were treated as described in Fig 1 and in Materials and Methods.

Finally, to understand if MF activates EGFR, we checked the phosphorylation level of EGFR on the residue Tyr1173 (Y1173), one of the first autophosphorylation sites upon EGFR dimerization and activation [31]. As shown in Fig 5, under all conditions, the total EGFR protein content retained unchanged, confirming the result by immunofluorescence in Fig 3. However, when compared to the sham, the level of the phospho-EGFR Tyr 1173 (p-EGFR 1173) was significantly elevated by 40.17% with MF exposure (Fig 5B, MF). A much more notable elevation was observed in the EGF treated samples, with an increase of 1276.19% (Fig 5B, EGF). This result suggested that MF might be able to activate the EGFR, though with a lower efficiency than EGF did. We also examined the EGFR phosphorylation level on the residue Ser1046/1047, whose phosphorylation regulates the inactivation process of EGFR through reducing its sensitivity to EGF [37]. It was found that the content of phospho-EGFR Ser1046/1047 (p-EGFR 1046/1047) in MF or EGF treated groups was also enhanced by 19.99% or 20.89%, respectively, when compared to the sham. These results imply that, while elevating EGFR activity, MF increases, but not depresses, the EGFR de-sensitivity to its ligand. Together with the results that MF decreased the PD inhibition on the TK domain of EGFR (Fig 4C, 4D and 4E) and the secretion of EGFR ligand TGF-alpha [13], MF is suggested to directly enhance EGFR activation at its catalytic site Tyr1173, depending on a mechanism similar to EGF but with a less efficiency.

In summary, here we confirmed that MF could induce EGFR clustering [38] by both immunofluorescence (Fig 3) and FRET (Fig 4). The results implied that MF induced EGFR clustering in a mechanism similar to EGF stimulation, which was relative to PD inhibition (Fig 4) [38, 65]. We also showed that MF activated EGFR (Fig 5) possibly due to EGFR autophosphorylation upon MF-induced clustering of EGFR monomers [31]. The findings that MF activated EGFR reminisced our previous results that MF induced cell migration and F-actin reorganization through up-regulating the F-actin cytoskeleton signal proteins of fascin, MLCK, and Arp2/3, which were all under the modulation of the EGFR pathway [13]. Moreover, MF-induced EGFR activation also modulated the MT instability, which will be described later.

### A significant role of the calcium signaling in MF effects on cytoskeleton

It was observed that a control level of the membrane EGFR clustering was still seen in serum-starved control cells free of EGFR ligands (Fig 4A and 4B, Sham), implying the EGFR was likely somehow transactivated by certain other element or elements. Therefore, it is interesting to inspect if there are certain signaling pathways other than MF induced EGFR clustering may also be on action upon MF exposure to activate EGFR.

The calcium signaling is well documented to cross talk with EGFR signaling: EGFR transactivation occurs upon evoking of the calcium signal pathway [39, 40], while Ca^2+^ entrance across cytosol membrane and [Ca^2+^]_i_ elevation happens in response to EGFR activation [40–42]. As abovementioned in this article, MF-induced effects were associated with calcium pathways, since simultaneous blockages of both EGFR and LTCC by PD and NIF not only fully erased the MF-induced changes on MT stability and cell morphology (PNM and PNE in Fig 1, and S2 Fig), but also seriously prevented the MF-induced redistribution and phosphorylation of EGFR proteins (PNM and PNE in Figs 3 and 5). Moreover, MF alone was reported to stimulate Ca^2+^ entering the cytosol [55–58], which likely plays a role in the mechanism of MF-induced effects. Therefore, we were very interested in the calcium pathway in its role in MF-induced cytoskeleton responses.

**Fig 6:**
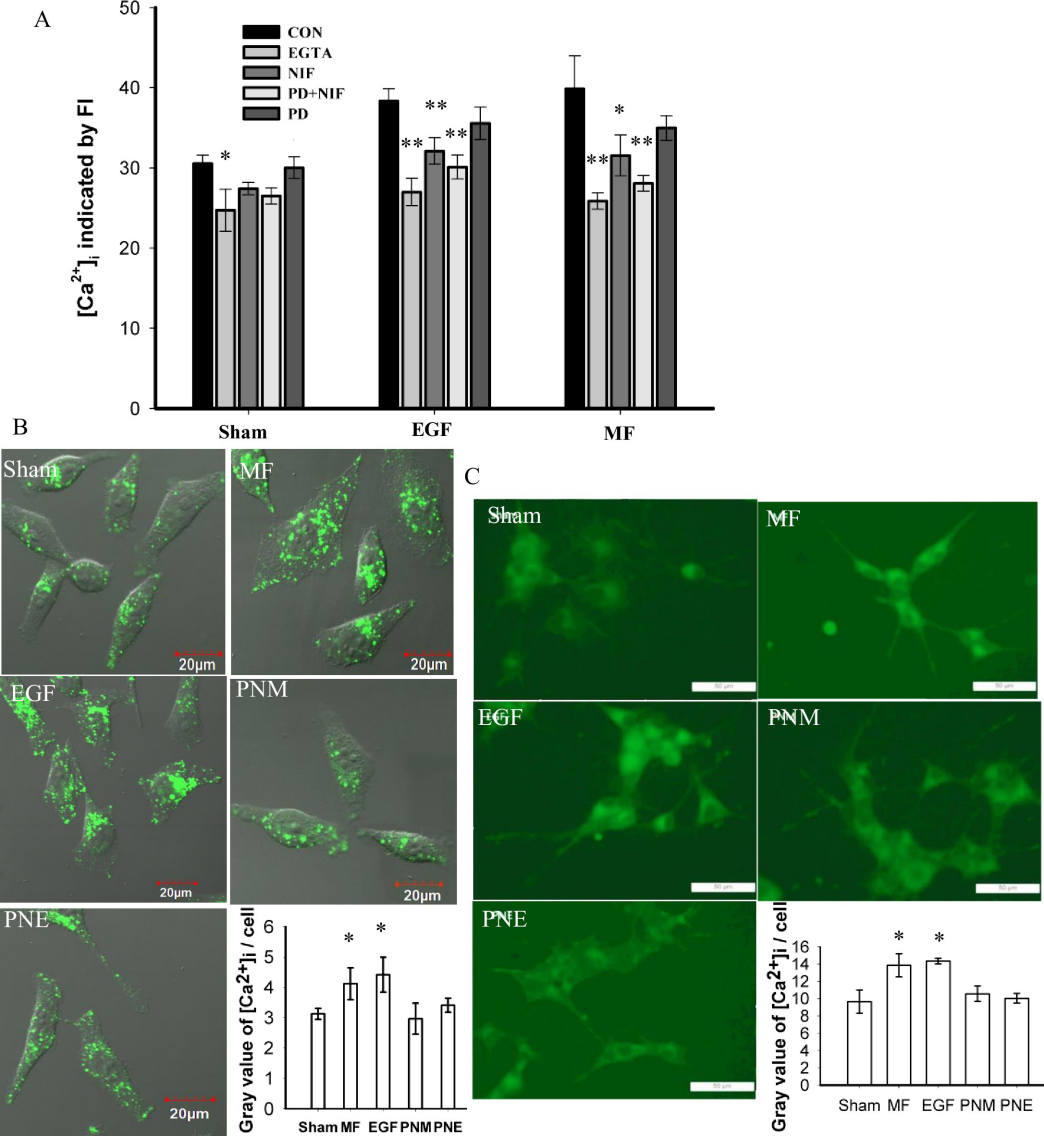
MF induced [Ca^2+^]_i_ elevation. A: [Ca^2+^]_i_ measured in FL cells by flow cytometric analyses. CON: control; EGTA: pretreated with as low as 1 μM [Ca^2+^]_o_ by EGTA chelation; NIF: pretreated with 0.02 mM NIF for 40 min; PD: pretreated with 1 μM PD for 2 h; PD+NIF: pretreated with 1 μM PD for 2 h and 0.02 mM NIF for 40 min; Sham: sham exposed for 30 min; MF: exposed to 0.4 mT 50 Hz MF for 30 min; EGF: treated with 100 nM EGF for 30 min. ** and *: P-value < 0.01 and < 0.05, respectively, when compared with the corresponding CON in the same group by Student’s t-test. B and C: [Ca^2+^]_i_ imaged by Fluo-4AM in FL (B) and PC12 (C) cells; the groups were treated as described in Fig 1 and in Materials and Methods. The horizontal line represents 20 μm in B and 50 μm in C; for each cell line, gray value of [Ca^2+^]_i_ in at least 100 cells of each condition was quantified in the bar chart, * and **: p-value < 0.05 and < 0.01, respectively, when compared with CON of the same group. Details of the analyzed cell number and repeat times were listed in S1 Table.

To elicit the influence of MF on calcium signaling, the intracellular calcium [Ca^2+^]_i_ was stained with fluo-3 AM and measured by flow cytometry (FCM). The [Ca^2+^]_i_ responds to the Ca^2+^ flux upon extracellular calcium ([Ca^2+^]_o_) alterations. As shown in Fig 6A, low [Ca^2+^]_o_ due to EGTA chelation resulted in a 19.08% decrease of mean FI from 30.56 ±1.04 in sham control (CON in Sham) to 24.73 ± 2.62 (EGTA in Sham). This effect was even more obvious in the MF and EGF groups. MF (CON in MF) and EGF (CON in EGF) similarly elevated [Ca^2+^]_i_ to 39.87 ± 4.09 and 38.34 ± 1.53, respectively, leading to a respective 30.46% and 25.46% increases relative to the CON in Sham (Fig 6A). These [Ca^2+^]_i_ elevation by MF and EGF was significantly, though not fully, eliminated by the pretreatment of NIF to inhibit LTCC (NIF). This implies that the increased [Ca^2+^]_i_ is possibly resulted from MF/EGF-triggered calcium influx, mainly through NIF-sensitive LTCC. With a lower efficiency than NIF did, the PD inhibition to EGFR alone also constrained the MF- and EGF-induced [Ca^2+^]_i_ elevation, indicating that the significant [Ca^2+^]_i_ elevations triggered by MF or EGF exposure partially relied on EGFR activation. However, PD+NIF double blockages resulted in a mean FI value of 31.54 ± 2.55 in EGF or 28.07 ± 0.98 in MF, proximate to the CON in Sham, but respectively 20.88% or 29.60% lower than CON in EGF or MF group (Fig 6A), implying PD+NIF pretreatment could almost fully, even excessively, block the MF- or EGF-induced [Ca^2+^]_i_ elevation.

The FCM results in FL cells were confirmed through the confocal method. Compared with the sham, [Ca^2+^]_i_ in FL was improved by 41.27% and 31.72% with MF and EGF treatment, respectively (Fig 6B). Similarly, the MF and EGF treatments also increased in PC12 cells the [Ca^2+^]_i_ by 43.80% and 48.83%, respectively (Fig 6C). In both cell lines, the pretreatments of PD+NIF prevented almost all the changes induced by MF or by EGF (PNE and PNM in Fig 6B and 6C). All these results suggested that MF elevated [Ca^2+^]_i_ in a manner depending on activation of both EGFR and LTCC.

**Fig 7:**
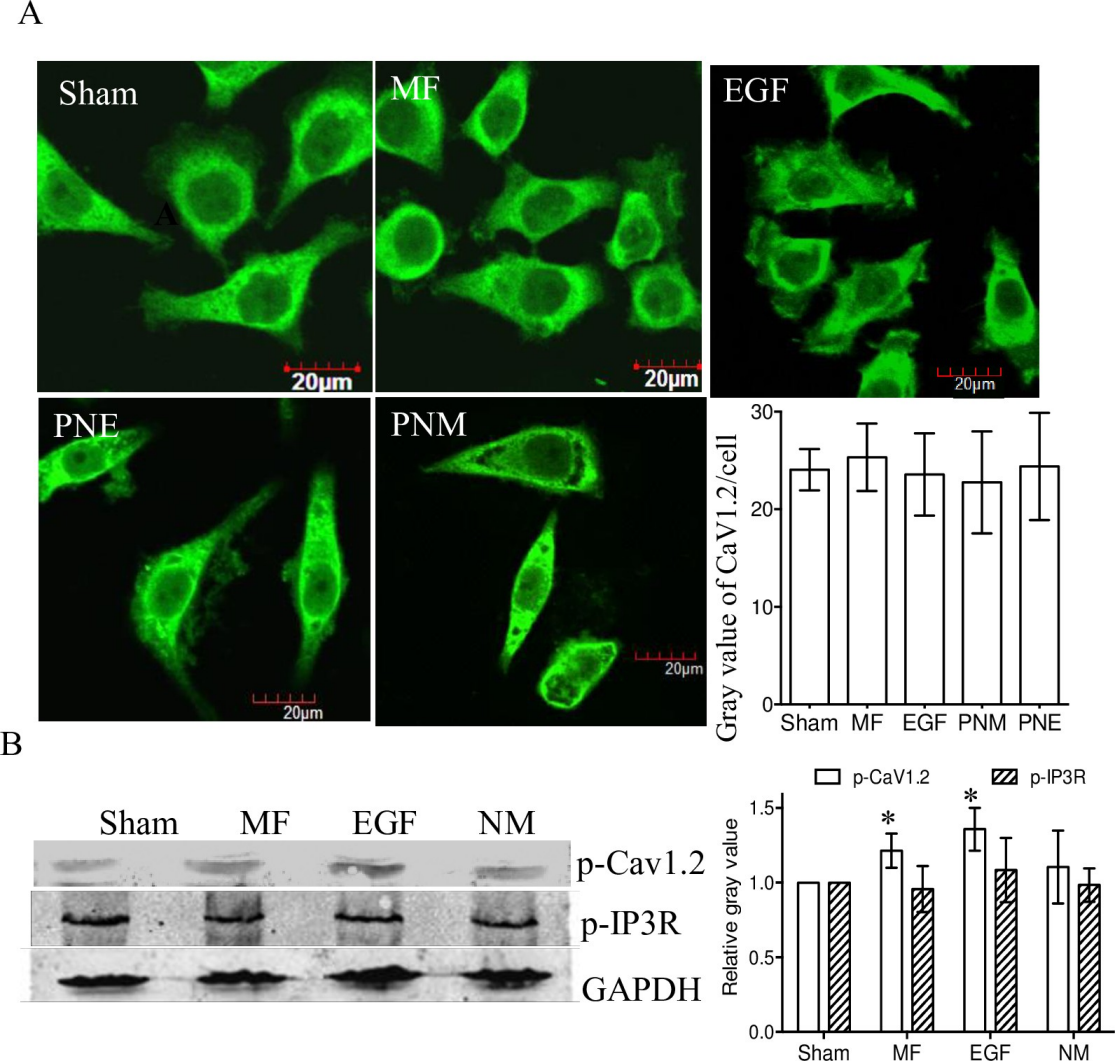
Effects of MF on CaV1.2 and IP3R. A: Immunofluorescence staining of CaV1.2 subunit of LTCC in FL cells. The groups were treated as described in Fig 1 and in Materials and Methods. The horizontal line represents 20 μm; average gray value of CaV1.2 per cell in at least 50 cells in each condition was quantified in the histogram; p-value > 0.05 when compared with Sham. B: Western blot assay (left) of p-CaV1.2 (top lane) and p-IP3R (middle lane) in FL cells; Sham: sham-exposed; MF: exposed to 0.4 mT MF for 30 min; EGF: treated with 100 nM EGF for 30 min; NM: pretreated with 0.02 mM NIF for 40 min, then exposed to 0.4 mT 50 Hz MF for 30 min. Relative gray value / content of the proteins to the Sham groups after normalized with the GAPDH was shown in the histogram (right). *: p-value < 0.05 by Student’s t-test when compared with Sham. Details of the analyzed cell number and repeat times were listed in S1 Table.

To confirm that LTCC indeed plays a role in allowing calcium influx through the channel, immunoblot experiments were conducted in FL cells. The results showed that the phosphorylation level of CaV1.2 (a key subunit of LTCC) was elevated by MF and EGF treatment (Fig 7B), though the total content was not significantly affected (Fig 7A and S3 Fig). Further analyses showed that, different from the EGF treatment, the MF mainly increased the phosphorylation level on the membrane CaV1.2, but not the proteins in cytosol (S3 Fig). These data suggests that the MF might activate the functional membrane, but not the internalized, LTCC to induce calcium influx [66]. One may argue that the [Ca^2+^]_i_ may also be elevated due to Ca^2+^ release from intracellular calcium pools. However, the activation of IP_3_R, a ubiquitous calcium channel mediating the release of intracellular calcium from endoplasmic reticulum in unexcitable cells [67], was not obviously detected upon MF or EGF treatment in FL cells (Fig 7B). This indicated that the observed evidences that MF or EGF induced [Ca^2+^] elevations are likely due to a Ca^2+^ influx from extracellular source through MF- or/and EGFR activated LTCC.

Researchers have revealed that MF could induce calcium intaking [55–58]. Here we confirmed that MF elevated cellular calcium level (Fig 6) through invoking calcium influx via activated LTCC (Fig 7). The calcium influx was relying on MF treatment, and at least partially dependent on EGFR activation (Fig 6), which reminisced the cross talks between the EGFR and calcium pathways [40–42].

### MF activated the MT network by evoking the signaling cascades involving both EGFR and calcium

#### The calcium pathways played a key role in MF-induced MT instability

**Fig 8:**
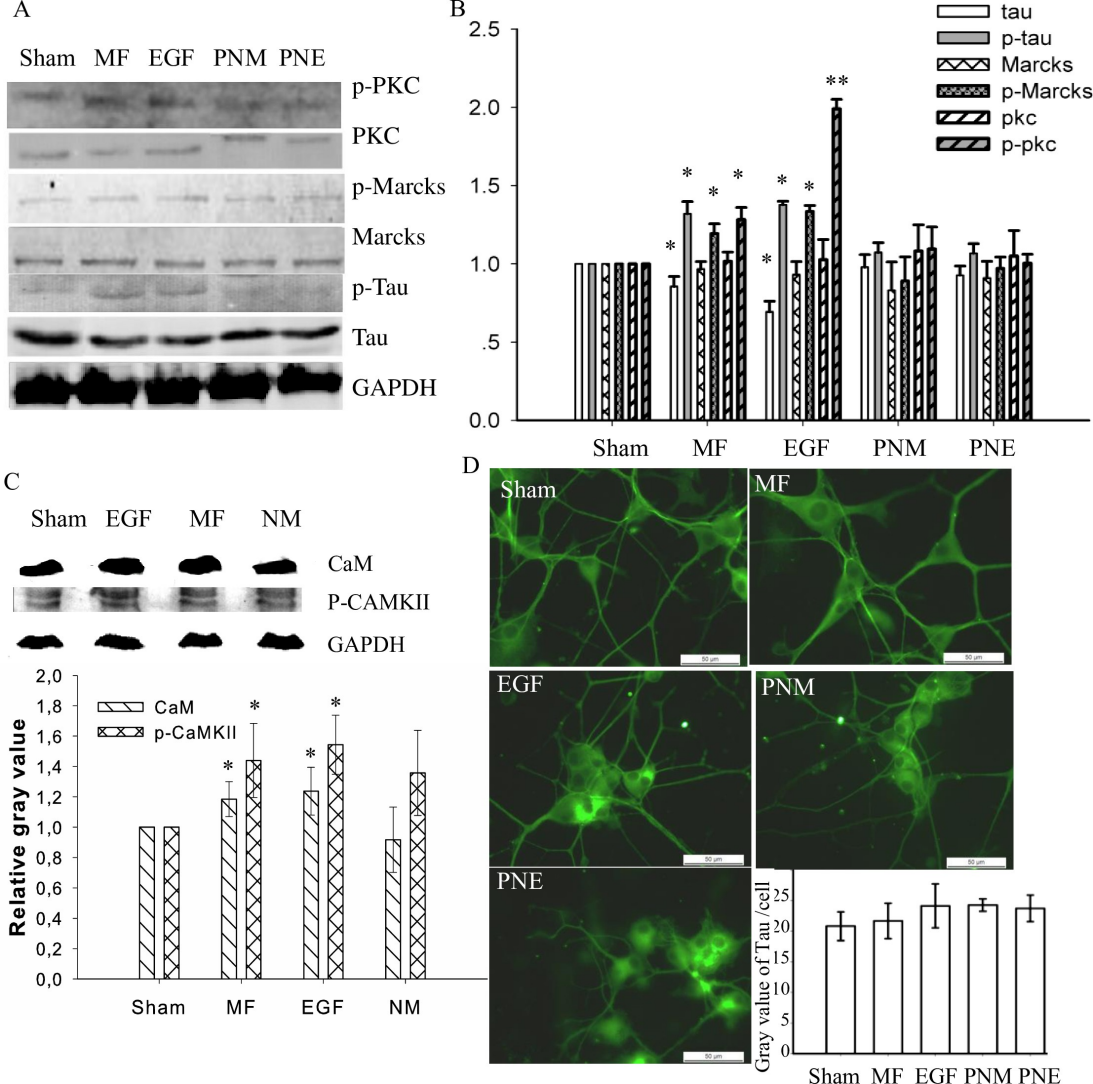
MF activated signaling proteins relative to both EGFR and calcium. A and B: Western blot of the indicated proteins (A) and the relative gray value / content of the proteins to the Sham groups after normalized with the GAPDH content (B); C: CaM and p-CAMKII by immunoblot (upper) and the quantification relative to the Sham after normalized to GAPDH (lower); D: Protein tau in PC12 cells by immunofluorescence. The groups in A, B, and D were treated as described in Fig 1 and in Materials and Methods; while the groups in C were treated as described in Fig 7B. * and ** in B and C: p-value < 0.05 and < 0.01 when compared with Sham by Student’s t-test, respectively. In D, the horizontal bars represent 50 μm; the average gray value per cell computed from at least 80 cells in each condition was shown in the histogram. The repeat time and analyzed cell number were described in detail in S1 Table.

**Fig 9:**
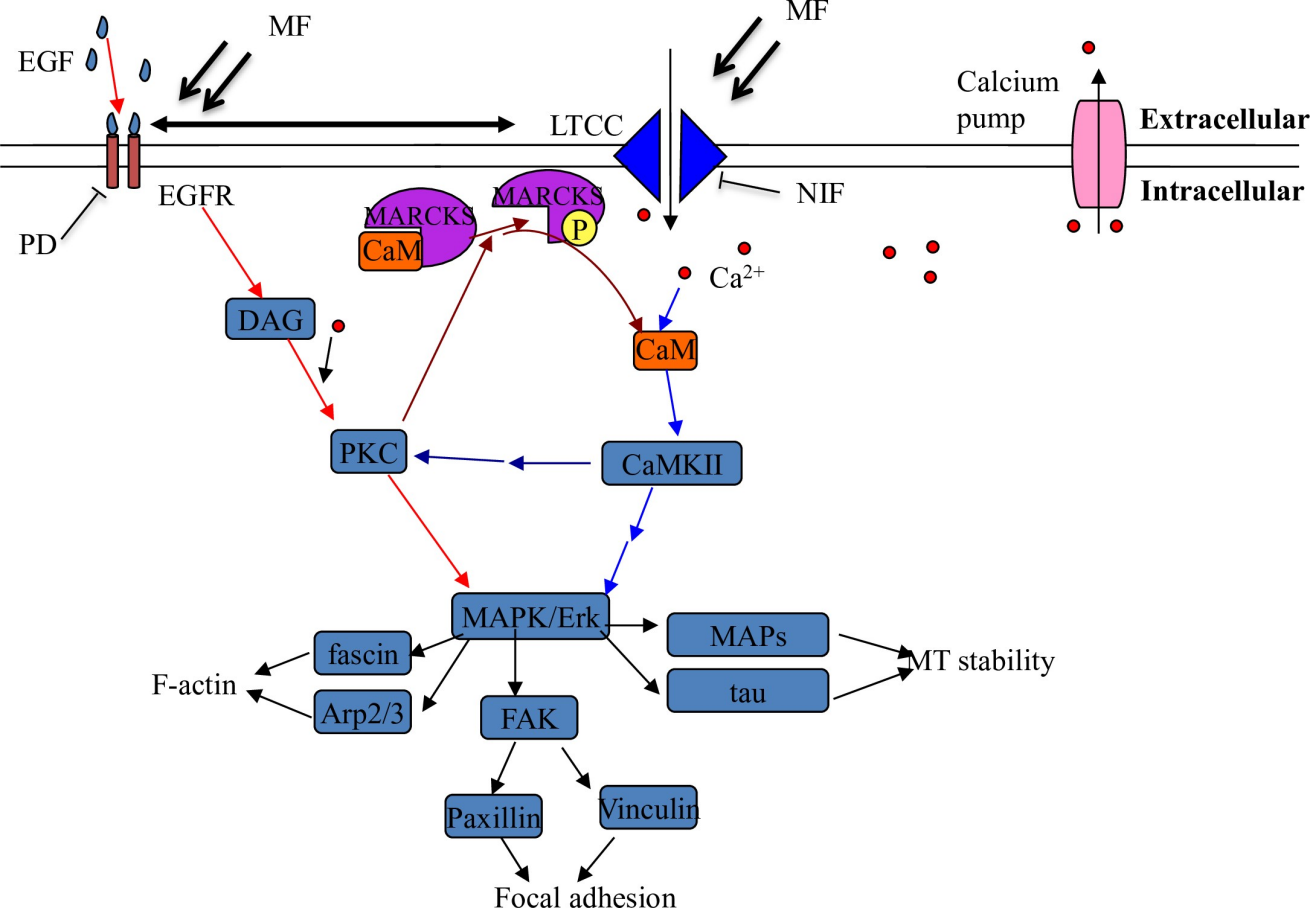
A schematic of how MF affects microtubule stability via EGFR and calcium pathways and the cross talks between the two. Arrows with blunt ends indicate inhibition relation. Red arrows: the EGFR-PKC-MAPK/Erk pathway [3–5]; blue arrows: the LTCC/CaMKII-MAPK/Erk pathway [46, 47]; dark red arrows: influence of the EGFR-PKC-MAPK/Erk pathway on the LTCC/CaMKII-MAPK/Erk pathway [43]; dark blue arrows: influence of LTCC/CaMKII-MAPK/Erk pathway on the EGFR-PKC-MAPK/Erk pathway [44–47]. The focal adhesions are also under the regulation of the MAPK/Erk pathway [50, 62].

Since the above results indicated that MF and EGF induced [Ca^2+^]_i_ elevation (Fig 6) through activating LTCC (Fig 7), we wonder if the downstreaming calcium signaling modulates MT instability. In cytosol, Ca^2+^ ions bind with CaM to form CaM/Ca^2+^ complex, which then regulates the phosphorylation of CAMKII [45]. Here, we observed that the phospho-CAMKII (p-CAMKII) level in FL cells was enhanced by 45.3% upon MF exposure. A similar increase of 49.5% was seen in the EGF treated sample (Fig 8C). The phosphorylated CaMKII protein was reported to directly activate MAPK/Erk pathway [3, 46, 47], and the phosphorylation level of MAPK/Erk was indeed increased upon the exposure of MF [68]. Activation of MAPK/Erk modulates MT through tau, a member of microtubule-associated proteins (MAPs) binding on MT to stabilize the fiber structure [24]. Upon phosphorylation, tau is released from the MT complex and MT becomes destabilized [20]. Here, both the protein and the phosphorylation levels of tau were investigated. It was found that the total tau protein content in FL cells was decreased by 14.45% or 30.70% with MF exposure or EGF treatment, respectively (Fig 8A and 8B). This decrease possibly relies on the proteasome and autophagy to degrade the phosphorylated tau [69]. In PC12 cells, no obvious decrease in total tau content was detected (Fig 8D). The level of phosphorylated tau (p-tau), however, conversely increased in FL cells by 32.00% and 37.80% in MF and in EGF treated groups, respectively. Therefore, MF is likely to destabilize MT network, in a way similar to EGF, by increasing tau phosphorylation level via Ca^2+^-CaMKII-MAPK/Erk-tau pathway (Fig 9).

#### MF destabilized MT through EGFR regulation

Besides the calcium pathway, it was also well documented that p-EGFR1173 was able to phosphorylate PKC [35, 36] to activate MAPK/Erk pathway[3, 46, 47] and thus to modulate cell motility [70]. We have found that the p-EGFR1173 level increased upon MF treatment (p-EGFR1173, Fig 5). Therefore, it is reasonable to investigate the influence of MF on PKC. The results showed that no change on the total PKC content was detected upon MF exposure (PKC, Fig 8A and 8B)). However, when compared to the sham, the phosphorylated PKC (p-PKC) level was greatly promoted by 28.24% in MF exposed cells. A similar but much more significant increase of 99.09% was observed in EGF treated cells, with negligible changes seen in NIF and PD pretreated cells (p-PKC Fig 8A and 8B), suggesting that MF activated PKC in a way similar to that of EGF, too. These results imply that the MF may induce MT instability through EGFR-PKC-MAPK/Erk-tau pathway (Fig 9).

EGFR activated by MF might exert effects on MT instability also through reinforcing calcium pathways. CaM is usually stored in Marcks sink, and can be released into cytosol upon PKC activation of Marcks [43]. MF exposure did not significantly change the total content of MARCKS (Fig 8A and 8B). However, compared to the sham, MF increased the level of p-MARCKS by 19.35%, similar but interior to a 33.45% increase in EGF treated group. Thus, it suggests that MF may amplify the activation of MARCKS, resulting in releasing more CaM free into cytosol [43]. The total CaM level was also seen 18.5% increase upon MF exposure (Fig 8C). By this way, MF invokes more free CaM and higher level of [Ca^2+^]_i_ (Fig 6), reinforcing the possibility of CAMKII phosphorylation (Fig 9).

The above results presented an interesting evidence to strongly suggest that the MF signaling transduction and exaggeration along the EGFR-PKC- MAPK/Erk [21–23] and the Ca^2+^-CaMKII- MAPK/Erk [44–47] pathways, and cross-talks occurrence between the two (Fig 9), showing that MF exposure could finally increase the tau phosphorylation level and destabilize the MT network though the signaling cascades. The tau hyperphosphorylation has been well known to contribute to the genesis of the Alzheimer’s disease [25, 26, 71, 72]. Abnormality of tau signaling has also been demonstrated to link with many other severe neuron degeneration diseases and brain disorders like frontotemporal lobar degeneration [73]. It is not yet clear if MF exposure is relative to the tauopathy, though, this work might shed a light on a possible mechanism by which MF was probably able to induce a weaker but more dynamic and flexible MT cytoskeleton, inferring the potential association between MF and the development of some relative illnesses.

#### MF destabilized MT network depending on both the EGFR and the calcium pathways

The MF induced MT instability involves both the EGFR and the LTCC pathways. One may wonder if the two pathways exert functions independently or sequentially, or there are cross-talks between the two, which collaboratively leads to cytoskeleton reorganization. Our results tend to the later possibility. There are several lines of clues indicated this hypothesis. First, NIF inhibition of LTCC alone could not fully restore the MF induced CaMKII phosphorylation (Fig 8C), and the MF induced morphological changes could be only fully erased by pretreatment of PD plus NIF, but not by either NIF or PD alone (S2 Fig and see the Fig 2 in [13]), indicating that these MF-induced changes depend on the activations of both the EGFR and calcium pathways, but not one single of the two. Simultaneous PD+NIF inhibition of both EGFR and LTCC also restored the effects of MF on MT reorganization (Fig 1A and 1B), cell reshaping (S2 Fig), FA redistribution (Fig 2), EGFR clustering (Fig 3) and activation (Fig 5), [Ca^2+^]_i_ elevation (Fig 6), and activation of their downstream signaling proteins like PKC, MARCKS, and tau (Fig 8), suggesting that all these MF-induced effects might rely on activation of both EGFR and LTCC pathways. Second, it has been reported that EGFR can be transactivated by the calcium [39, 40], while EGF can enhance [Ca^2+^]_i_ [40–42]. Here, we’ve also found that MF/EGF elevated [Ca^2+^]_i_ level (CON in MF or EGF, Fig 6A). PD inhibition of EGFR alone could significantly depressed the MF-induced [Ca^2+^]_i_ elevation (PD in MF, Fig 6A), suggesting that the [Ca^2+^]_i_ elevation upon MF exposure partially attributes to EGFR transactivation of the calcium channel. Calcium influx is documented to phosphorylate MAPK/Erk via activation of EGFR, which can be blocked by LTCC inhibition [39, 40]. Last but not the least, there are strong crosslinks between the two pathways. The EGFR activated PKC is able to increase p-MARCKS to release CaM from MARCKS sink (Figs 8 and 9) [43], thus reinforces the Ca^2+^-CaMKII-MAPK/Erk-tau signaling (Fig 9) [44–47]. Meanwhile, the Ca^2+^ and p-CaMKII in turn could contribute to PKC phosphorylation [44], probably therefore to strengthen the PKC-MAPK/Erk pathway (Fig 9). Taken together, we showed intensive crosslinks between the EGFR and LTCC-Ca^2+^ pathways, through which MF exerted its effects on MT instability and reorganization, but could neither declare the sequence or order of activation of the two pathways, nor separate the functions of the two pathways on MT reorganization.

## Conclusions

Taking together with our previous results [13], we showed that the MF, in a way similar to EGF but independent of the EGFR ligand, provoked both F-actin and MT cytoskeleton into a migrating/ready-to-migration state in epithelial and neuron cells. We report in this paper as the first-hand but primary evidence that the MF exposure induces the MT reorganization via activation of both the EGFR-PKC-MAPK/Erk and the Ca^2+^-CaMKII-MAPK/Erk pathways, and cross talks between them (Fig 9). Meanwhile, we have also shown that MF induces the formation and redistribution of focal adhesions, possibly through proteins paxillin and FAK, which are under the regulation of EGFR [62]. Relying on these EGFR and Ca^2+^ pathways and the crosstalk between them, the MF signal is transformed into biological signal and transmitted along the signal transduction cascades. In this manner, the effects of MF could be exaggerated and might finally exert influence to activate the overall F-actin- and MT-cytoskeleton, to trigger the growth of protrusional structures and focal adhesions, and to improve the cell migration in different cell lines.

## Materials and methods

### Reagents

Purified human-derived EGFR (#E2645), native EGF (#E9644), anti-phospho-CAMKII antibody (pThr287) (#SAB4504607), anti-β-Tubulin antibody (#T8328), Antipyrylazo III (APIII, #089F0935), and Nifedipine (NIF, #N7634) were obtained from Signa-Aldrich. The anti-EGFR antibody (#2239), phospho-EGF Receptor (Tyr1173) (53A5) antibody (#4407), phospho-EGF Receptor (Ser1046/1047) antibody (#2238), phospho-PKC (pan) (βII Ser660) antibody (#9371), anti MARCKS (D88D11) antibody (#5607), phospho-MARCKS (Ser152/156) antibody (#2741), anti-tau (tau46) antibody (#4019), phospho-tau (pSer404) antibody (#T7444), anti-vinculin antibody (#4650), anti- phospho-IP3R (Ser1756) (#3760), anti-mouse secondary antibody conjugated to Alexa Fluor 488 (#4408) and to Alexa Fluor 555 (#4409), anti-rabbit secondary antibody conjugated to Alexa Fluor 488 (#4412) and to Alexa Fluor 555 (#4413) were purchased from Cell Signaling Technology. The EGF-Alexa 488 (#E13345) and EGF-Alexa 555 (#E35350) were from Life Technologies. The anti-PKC beta 2 antibody [Y125] (#ab32026) was purchased from Abcam, while the anti-GAPDH antibody (#AG019) and RIPA lysis buffer (Cat: P0013B) were obtained from Beyotime. The protease inhibitor cocktail tablets (#04693116001) were from Roche. Neuron growth factor (NGF, #556-NG) was from R&D systems. PD153035 (#234490) was purchased from Calbiochem. Goat anti-rabbit IgG (H + L) (IRDye 800CW Conjugated) (#926–32211) and goat anti-mouse IgG (IRDye 800CW Conjugated) (#926–32210) were purchased from LI-COR biosciences. PhosphoSafe Extraction Reagent (#71296) was from Merck Millipore. The Fluo-3 AM (#F1242) was bought from Molecular Probes, while the Fluo-4 AM (#F14201) was from Invitrogen. Anti-CaV1.2 (#NBP1-42817) was from Novus Biologicals. Anti-phospho-CaV1.2 (Ser1928) (#A010-70) was from Badrilla.

### Cell culture, MF exposure, and experimental conditions

Human amniotic epithelium FL cells (a kind gift from Dr. Qunli Zeng, Zhejiang University) were cultured in minimum essential medium (MEM, GIBCO), supplied with 15% fetal bovine serum (FBS, GIBCO) [13]. The pheochromocytoma 12 (PC12, a kind gift from Dr. Yan-Ai Mei, Fudan University) cells were maintained in the Kaighn's modification of Ham's F-12 medium (F12K, GIBCO), supplied with 10% FBS and 5% horse serum (GIBCO), or with 2% FBS, 2% horse serum, and 50μg/mL neuron growth factor (NGF) when inducing differentiation. Both cell lines were maintained with streptomycin and penicillin (Beyotime, final concentration: 100 unites/mL each) in 37°C incubator with 5% CO_2_ and humid atmosphere. The FL cells at the 5th-7th generations and the PC12 cells with NGF treatment for 1 week, unless otherwise mentioned, were used as the experimental substrates.

The MF exposure system was already described in [13]. Considering that the international commission on non-ionizing radiation protection (ICNIRP) safety guideline for power frequency MF suggests a 0.2 mT limit intensity for the public and 1 mT for professionals [74], the MF finally used in this study was 0.4 mT, 50 Hz. The sham-exposed cells were placed in the same condition as in the MF group, except with the MF system in off-position [38].

The cells were divided into different groups: Sham was cells sham-exposed for 30 min; MF was treated only with 0.4 mT MF for 30 min; EGF was treated with 100 nM EGF for 30 min in sham condition; PD and NIF meant pretreatment of 1 μM PD for 2 h and 20 μM NIF for 40 min before sham exposure, respectively; and PNM or PNE were MF or EGF groups, respectively, with pretreatment of both 1 μM PD for 2 h and 20 μM NIF for 40 min. In FRET experiments (seen in the below section), sometimes 100 nM EGF was added while MF exposure for 30 min (EGF+MF).

### Fluorescence resonance energy transfer experiments

Fluorescence resonance energy transfer (FRET) has been employed in detecting EGFR clustering [63]. Here the concentration ratio of the donor/acceptor dyes was set to be 1/2 to ensure that 80% of the donor particles have at least one acceptor partner [63], and the signal changes at the donor’s emission peak (DEP) were computed as the FRET signal.

For the FRET experiments within cells, the FL cells were pretreated and sham/MF exposed as aforementioned, then placed on ice bed, and rinsed with ice-cold PBS three times before fixed with 4% paraformaldehyde for 3 h at 4°C. After rinsing by PBS for 3 times, the cells were collected by scraper and rinsed with PBS for another 3 times by spinning at 1000 g at room temperature (RT). For each sample, around 2–3 million cells were resuspended in 5 mL PBS with intensively pipetting and vortex to diffuse into single cell state, and then the cell suspension was split into 1mL aliquots as subgroups. The cells were incubated overnight at 4°C in dark, with 300 nM native EGF as background in the first aliquot, 100 nM of EGF-alexa 488 (Excitation/Emission (Ex/Em): 495/519 nm) plus 200 nM of native EGF as donor in the second aliquot, 200 nM EGF-Alexa 555 (Ex/Em: 555/565 nm) plus 100 nM of native EGF as acceptor in the third aliquot, and 100nM EGF-alexa 488 plus 200 nM EGF-Alexa 555 as FRET in the fourth aliquot. After rinsed with cold PBS 3 times by centrifugation at 1000 g for 10 min, a 1 mL (1x10^5^/mL) cell suspension for each subgroup was applied to measure the fluorescence intensity (FI) signals. The repeat times of the experiments were listed in the S1 Table.

As for the experiments with purified protein, EGFR monomer solution was prepared with PBS, and the final concentration was 5 μg/mL [38]. As the cell samples, after the indicated pretreatments and MF/sham exposure, the samples were carefully split into 4 groups and were then labeled as background, donor, acceptor, and FRET with the EGF probes. To get rid of the effects of EGF on EGFR oligomerization, the samples were also labeled with antibodies [75]. The anti-EGFR antibody (1: 200) was added 30 min before the secondary antibody. No secondary antibody, only secondary antibody conjugated with alexa-488 (1:400), only secondary antibody conjugated with alexa-555 (1:200), or both antibodies were added in the group of background, donor, acceptor or FRET, respectively. The repeat time m and total parallel number n of the experiments were listed in the S1 Table.

The FI signal was collected by a fluorescence spectrophotometer (HORIBA JY FM-4 from Hitachi, Japan) with excitation spectrum of 495 nm. With a filter reducing signals < 510 nm, FI was collected in the spectrum of 500–600 nm. The FI was normalized by the corresponding background sample, and then the average FI within 515–525 nm was calculated as the DEP FI to be used in the analyses. Finally, the percentage of DEP FI decrease relative to the donor+accepter was calculated as the parameter of FRET. In the [PD] dependent FRET experiments, the samples were pretreated with the indicated [PD] for 2 h, and the IC_50_ and K_d_ were calculated from the FI decrease percentages at DEP with Hill-Plot function in Sigmaplot software.

### Estimating [Ca^2+^]_i_ by flow cytometry analysis

Measurement of cytosolic Ca^2+^ concentration ([Ca^2+^]_i_) was described elsewhere [76] with minor modifications. In this study, we used Fluo3-AM, which has been shown as a reliable membrane penetrated dye to detect changes in cytosolic calcium [77, 78], to stain calcium ions.

To modulate the [Ca^2+^] in the extracellular matrix ([Ca^2+^]_o_), the [Ca^2+^]_o_ was set to 1 μM, 1 mM or 10 mM by EGTA at pH 7.1. The [Ca^2+^]_o_ was monitored and calibrated optically using Antipyrylazo III (APIII) in serial standard [Ca^2+^] buffers of 0.1–11 mM to determine [Ca^2+^]_o_ [79].

FL cells were divided into 5 groups, with CON as control, with outside cell calcium [Ca^2+^]_o_ level as low as 1 μM by EGTA chelating in EGTA group, with pretreatment of 0.02 mM NIF for 40 min (NIF), or with 1 μM PD for 2 h (PD), or with both 0.02 mM NIF for 40 min and 1 μM PD for 2 h (PD+NIF). Cells of each group were separated into 3 subgroups, and treated with sham, 100 nM EGF, or 0.4 mT 50 Hz MF for 30 min, respectively. After 3 times of rinse with PBS, cells were incubated with 24.8 μg/ml Fluo-3/AM (Ex/Em: 488/525 nm) for 40 min at 37°C in the dark, followed with PBS rinse for 3 times. A 0.5 ml (1×10^5^/ml) cell suspension was collected for each condition for flow cytometric measurements (FACScan, Becton Dickson, Franklin Lakes, NJ, USA). The mean FI of each measurement was analyzed and normalized by cell number using Cell Quest software (FACScan, Becton Dickson Company). The experiments were repeated as described in S1 Table.

### Immunofluorescence

The FL and PC12 were seeded on glass coverslips in 6-well plates at 1x 10^4^/mL and divided into the aforementioned 5 groups. After indicated pretreatments and the sham/MF exposure, the cells were rinsed with PBS for 3 times, 5 min each time, and then fixed by 4% paraformaldehyde for 10 min at room temperature (RT), followed by PBS rinse for 3 times, 5 min each time. Then the cells were treated with 0.22% Triton X-100 for 10 min at RT, washed with PBS 3 times, 5 min each time. Corresponding primary antibody and secondary antibody were applied in sequence to label each target protein according to the manufacture’s direction, both for 1 h at RT, secondary antibody in dark, with 3 times of 5 min PBS rinse in between. To stain Ca^2+^, the cells were manipulated as abovementioned, except to skip the Triton X-100 treatment and to incubate with Flou-4 AM (Ex/Emi: 494/506 nm) at a final concentration of 2.2 μmol/L at 4°C in dark overnight. After labeling, the cells were rinsed by PBS for 3 times, 5 min per time. The coverslips were sealed on slides and then observed under the laser scanning confocal microscope or fluorescence microscope. For each labeled protein and each condition, the repeat time m, total parallel number n, and analyzed cell number were listed in S1 Table.

The amount of fluorescence in each cell was collected as average gray value per cell with the software ImageJ 1.46 (NIH, http://rsb.info.nih.gov/ij/download.html). The average gray value was computed based on data from 35–70 cells for each labeled protein in each condition (see details in S1 Table), and normalized to the sham, so that no standard deviation was in sham group.

### Western blotting assays

Cells were treated as abovementioned, then immediately placed on ice and rinsed with cold PBS once. After adding RIPA lysis buffer (or PhosphoSafe extraction reagent for the samples to detect protein phosphorylation levels) with protease inhibitor according to manufacturer’s direction, cells were scraped and centrifuged at 13,000 g at 4°C for 10 min to remove the cell debris. Each target protein was marked with its corresponding primary and secondary antibodies. The gray values of the protein bands were obtained by ImageJ and normalized to the reference protein GAPDH and the sham group. The repeat time m and total parallel number n were listed in S1 Table.

### Statistics

The data was normalized to the sham, and was shown as mean or mean ± standard deviation (sd, shown as error bar), except the sham group, which was normalized as 1. One exception was in Fig 4D, where the data was shown as Ln(mean) and the error bar represented Ln(mean-sd). Student’ t-test was used to test the significance of differences relative to the sham, unless otherwise specially indicated; significant difference and extremely significant difference were inferred as * and ** if p-value < 0.05 or < 0.01, respectively. The parallel sample number and/or analyzed cell number were listed in S1 Table.

## Supporting information

S1 Fig Effects of MF on the differentiation of PC12 cells. A: Differentiating cells on the first (left), third (middle), and seventh (right) day after adding the NGF to induce PC12 differentiation. Upper lane: Sham; lower lane: exposed to 50 Hz, 0.4 mT MF; the horizontal bar represents 200 μm. B: Percentage of differentiated cells from A. The differentiated cells were defined as those with axon length longer than the cell body diameter. At each point, at least 200 cells were examined; repeat time n = 3; *: p-value < 0.05 when compared to the Sham by Student’s t-test.
(PDF)

S2 Fig MF induced protrusions in FL cells that were not totally rescued by inhibiting EGFR. FL cells were sham (A) or exposed to 0.4 mT 50 Hz MF (B) or treated with 100 nM EGF (F) for 30 min; or FL cells were pretreated with 1 μM PD for 2 h (C) or 20 μM NIF for 40 min (D) or with both (E) before MF exposure (C-E) or EGF treatment (G) for 30 min. Arrow: appearance of filopodia, arrowhead: lamellipodia. A-D and F was from [13].
(PDF)

S3 Fig Effects of MF on CaV1.2 and IP3R. A: Contents of CaV1.2 in FL cells by Western blot (left) and the relative gray value to the Sham group after normalized with the GAPDH content (right); Sham: sham-exposed; MF: exposed to 0.4 mT MF for 30 min; p-value > 0.05 when compared with Sham by Student’s test. B: p-CaV1.2 content in the membrane and cytoplasm part of FL cells. The cytoplasm and membrane parts of the FL cells were separated and the p-CaV1.2 content in each part was examined by Western blot and the quantification from 3 repeats was shown in the histogram. *: p-value < 0.05 when compared to the Sham by Student’s test.
(PDF)

S1 Table Repeat times and analyzed cell numbers. (PDF)
