# Supplementary material for: Weak power frequency magnetic fields induce microtubule cytoskeleton reorganization depending on the epidermal growth factor receptor and the calcium related signaling
Source: PLoS One. 2018 Oct 12;13(10):e0205569. doi: 10.1371/journal.pone.0205569 (PMC6185734; doi:10.1371/journal.pone.0205569)
Supplement: S1 Fig — A: Differentiating cells on the first (left), third (middle), and seventh (right) day after adding the NGF to induce PC12 differentiation. Upper lane: Sham; lower lane: exposed to 50 Hz, 0.4 mT MF; the horizontal bar represents 200 μm. B: Percentage of differentiated cells from A. The differentiated cells were defined as those with axon length longer than the cell body diameter. At each point, at least 200 cells were examined; repeat time n = 3; *: p-value < 0.05 when compared to the Sham by Student’s t-test. (PDF) [file pone.0205569.s001.pdf]

S1 Fig. Effects of MF on the differentiation of PC12 cells

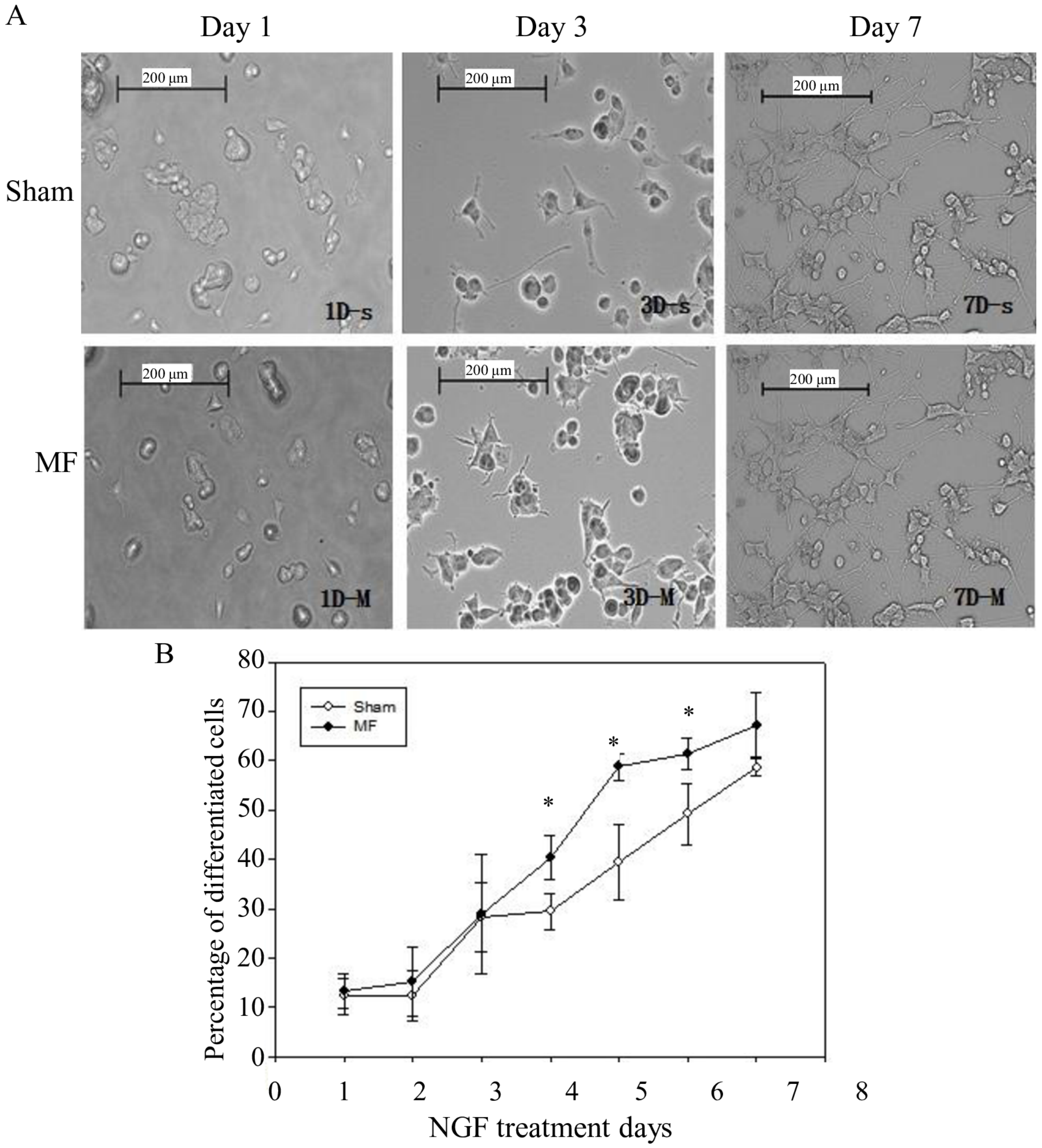

S1 Fig

S1 Fig. Effects of MF on the differentiation of PC12 cells. A: Differentiating cells on the first (left), third (middle), and seventh (right) day after adding the NGF to induce PC12 differentiation. Upper lane: Sham; lower lane: exposed to 50 Hz, 0.4 mT MF; the horizontal bar represents 200 μm. B: Percentage of differentiated cells from A. The differentiated cells were defined as those with axon length longer than the cell body diameter. At each point, at least 200 cells were examined; repeat time n=3; *: p-value < 0.05 when compared to the Sham by Student's t-test.

Methods: The PC12 cells were seeded on glass cover slips in 6-well plates at 1x 10^4/mL in presence of 50μg/mL NGF and continuously sham/MF exposed. Pictures were taken on the 1st, 3rd, and 7th day. For each cell, the axon length and the cell body diameter were measured in ImageJ. The differentiated cells are defined as those with axon length longer than the cell body diameter. The experiments were repeated 3 times with 6 parallel samples in all, and for each condition, more than 200 cells were analyzed.
