# Supplementary material for: Weak power frequency magnetic fields induce microtubule cytoskeleton reorganization depending on the epidermal growth factor receptor and the calcium related signaling
Source: PLoS One. 2018 Oct 12;13(10):e0205569. doi: 10.1371/journal.pone.0205569 (PMC6185734; doi:10.1371/journal.pone.0205569)
Supplement: S2 Fig — FL cells were sham (A) or exposed to 0.4 mT 50 Hz MF (B) or treated with 100 nM EGF (F) for 30 min; or FL cells were pretreated with 1 μM PD for 2 h (C) or 20 μM NIF for 40 min (D) or with both (E) before MF exposure (C-E) or EGF treatment (G) for 30 min. Arrow: appearance of filopodia, arrowhead: lamellipodia. A-D and F was from [13]. (PDF) [file pone.0205569.s002.pdf]

S2 Fig. MF induced protrusions in FL cells that were not totally rescued by inhibiting EGFR

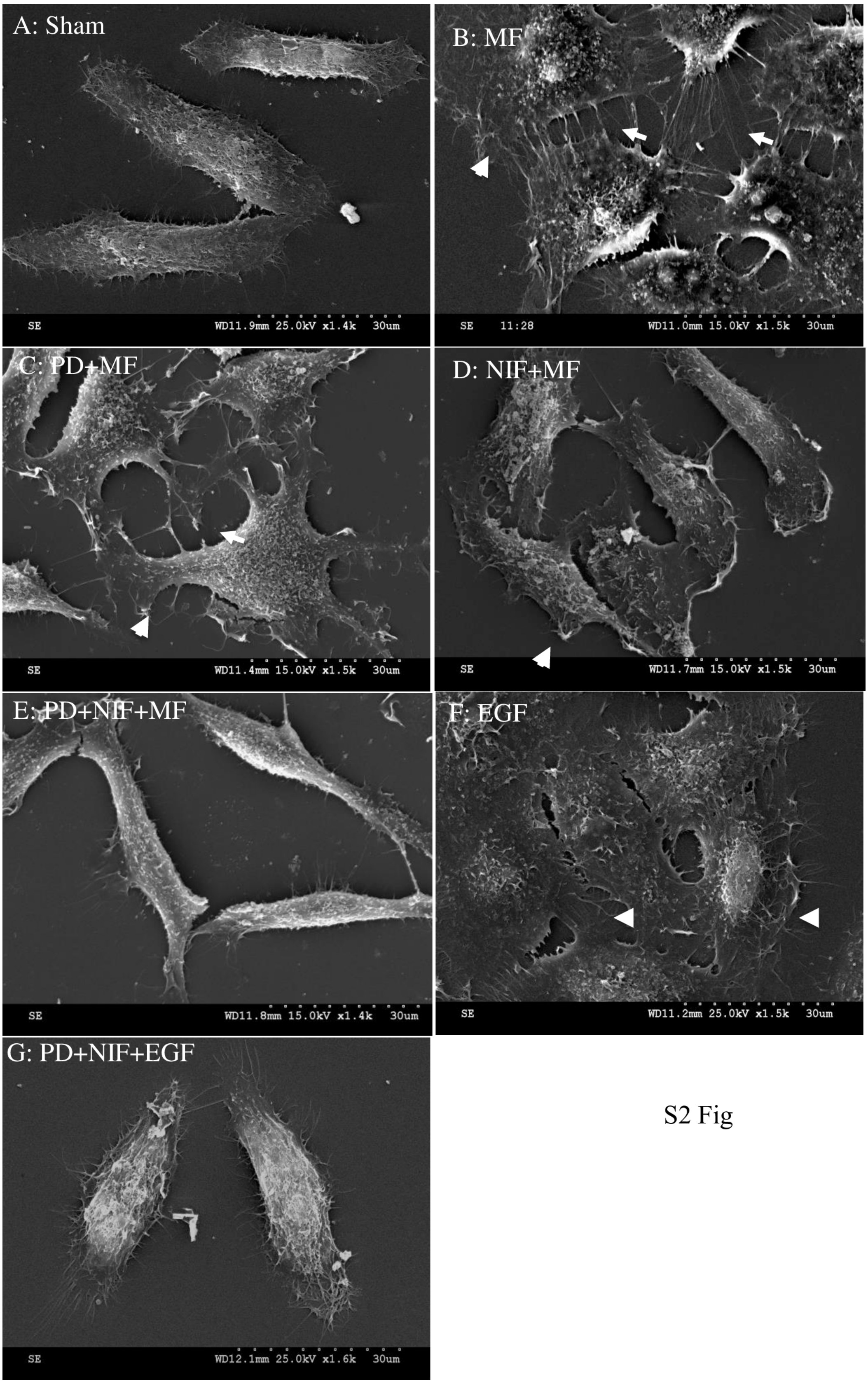

S2 Fig

S2 Fig. MF induced protrusions in FL cells that were not totally rescued by inhibiting EGFR. FL cells were sham (A) or exposed to 0.4 mT 50 Hz MF (B) or treated with 100 nM EGF (F) for 30 min; or FL cells were pretreated with 1 μM PD for 2 h (C) or 20 μM NIF for 40 min (D) or with both (E) before MF exposure (C-E) or EGF treatment (G) for 30 min. Arrow: appearance of filopodia, arrowhead: lamellipodia. A-D and F was from [1].

References

1. Wu X, Cao MP, Shen YY, Chu KP, Tao WB, Song WT, et al. Weak power frequency magnetic field acting similarly to EGF stimulation, induces acute activations of the EGFR sensitive actin cytoskeleton motility in human amniotic cells. PloS one. 2014;9(2):e87626. doi: 10.1371/journal.pone.0087626. PubMed PMID: 24505297; PubMed Central PMCID: PMC3914819.
