# Supplementary material for: Weak power frequency magnetic fields induce microtubule cytoskeleton reorganization depending on the epidermal growth factor receptor and the calcium related signaling
Source: PLoS One. 2018 Oct 12;13(10):e0205569. doi: 10.1371/journal.pone.0205569 (PMC6185734; doi:10.1371/journal.pone.0205569)
Supplement: S3 Fig — A: Contents of CaV1.2 in FL cells by Western blot (left) and the relative gray value to the Sham group after normalized with the GAPDH content (right); Sham: sham-exposed; MF: exposed to 0.4 mT MF for 30 min; p-value > 0.05 when compared with Sham by Student’s test. B: p-CaV1.2 content in the membrane and cytoplasm part of FL cells. The cytoplasm and membrane parts of the FL cells were separated and the p-CaV1.2 content in each part was examined by Western blot and the quantification from 3 repeats was shown in the histogram. *: p-value < 0.05 when compared to the Sham by Student’s test. (PDF) [file pone.0205569.s003.pdf]

S3 Fig. Effects of MF on CaV1.2

A

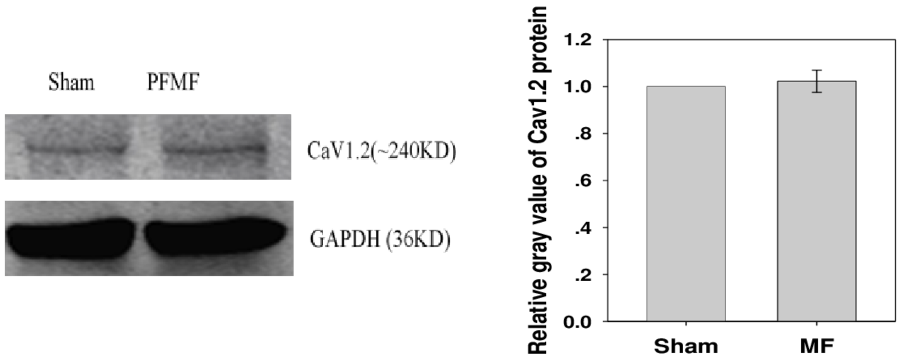

B

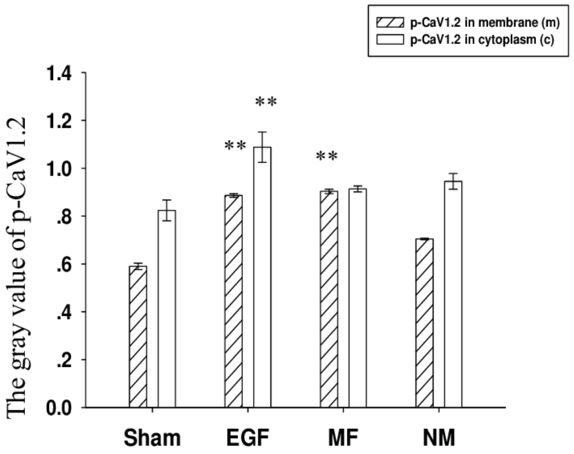

S3 Fig

S3 Fig. Effects of MF on CaV1.2. A: Contents of CaV1.2 in FL cells by Western blot (left) and the relative gray value to the Sham group after normalized with the GAPDH content (right); Sham: sham-exposed; MF: exposed to 0.4 mT MF for 30 min; p-value > 0.05 when compared with Sham by Student's test. B: p-CaV1.2 content in the membrane and cytoplasm part of FL cells. The cytoplasm and membrane parts of the FL cells were separated and the p-CaV1.2 content in each part was examined by Western blot and the quantification from 3 repeats was shown in the histogram.

Methods: The experiment was carried out as those western blot experiments described in Materials and Methods, except that the Membrane and Cytosol Protein Extraction Kit (#P0033, Beyotime) was used to extract proteins separately from cell membrane and cell cytosol.
