# Supplementary material for: Weak power frequency magnetic fields induce microtubule cytoskeleton reorganization depending on the epidermal growth factor receptor and the calcium related signaling
Source: PLoS One. 2018 Oct 12;13(10):e0205569. doi: 10.1371/journal.pone.0205569 (PMC6185734; doi:10.1371/journal.pone.0205569)
Supplement: S1 Table — (PDF) [file pone.0205569.s004.pdf]

## S1 Table. Repeat times and analyzed cell numbers.

| | Condition | Number of repeat (m) | Number in repeat (n) | Number of analyzed cells |
|---|---|---|---|---|
| **SEM (S2 Fig)** | NIF | 3 | 6 | 12 |
| | Each of rest | 6 | 12 | 34 |
| **MT in FL/PC12 (confocal, Fig1)** | sham | 04/03 | 06/05 | 39 / 42 |
| | MF | 04/03 | 06/06 | 45 / 31 |
| | EGF | 04/03 | 05/05 | 39 / 42 |
| | Each of rest | 04/03 | 04/04 | 40 / 35 |
| **PC12 differentiation (microscopy, S1 Fig)** | Sham | 3 | 6 | >200 |
| | MF | 3 | 6 | >200 |
| **EGFR clustering (FRET, antibody, Fig4)** | Each of all | 3 | 6 | / |
| **EGFR clustering (FRET, EGF, Fig 4)** | Each of all | 3 | 6 | / |
| **EGFR in FL/PC12 (confocal, Fig 3)** | sham | 6 | 11 | >70 |
| | MF | 5 | 9 | >70 |
| | Each of rest | 3 | 6 | >50 |
| **EGFR WB (Fig 5)** | Each of all | 3 | 5 | $\sim 2\times10^6$ |
| **Ca2+ in FL/PC12 (confocal, Fig 6)** | Sham | 04/03 | 07/06 | >45 |
| | MF | 04/03 | 08/06 | >45 |
| | Each of rest | 04/03 | 05/04 | >35 |
| **CaV1.2 (confocal, Fig 7)** | Each of all | 4 | 8 | >50 |
| **p-CaV1.2/p-IP3R WB (Fig 7)** | Each of all | 3 | 6 | $\sim 3\times10^6$ |
| **CaV1.2 WB (Fig 7)** | Each of all | 4 | 7 | $\sim 3\times10^6$ |
| **p-PKC/PKC/p-Marcks/Marcks/p-Tau/Tau/GAPDH WB (Fig 8)** | Each of all | 3 | 6 | $\sim 3\times10^6$ |
| **CaM/p-CAMKII (Fig 8)** | Sham | 3 | 4 | $\sim 2\times10^6$ |
| | Each of rest | 3 | 6 | $\sim 3\times10^6$ |
| **Tau (confocal, Fig 8)** | Each of all | 3 | 6 | >60 |
| **Vinculin (confocal, Fig 2)** | Each of all | 3 | 6 | >60 |
| **$[Ca^{2+}]_i$ (Flow cytometry, Fig 6)** | Sham | 6 | 30 | $\sim 3\times10^5$ |
| | Each of rest | 3 | 10 | $\sim 1\times10^5$ |

~: about.

m: the number of independent experimental trials carried out for each biological target.

n: the number of total parallel samples.
